# Study on fracture of coal samples with different fracture angles under microbial environment

**DOI:** 10.1371/journal.pone.0333227

**Published:** 2025-11-06

**Authors:** Wen Wang, Yuxiang Song, Daping Xia, Xiaowei Lu, Songsong Guan, Chuanjiu Zhang, Yiwen Ju

**Affiliations:** 1 College of Energy Science and Engineering, Henan Polytechnic University, Jiaozuo, Henan, China; 2 Coal safety production and clean and efficient use of the province to build a collaborative innovation center, Jiaozuo, Henan, China; 3 College of Resources and Environment, Henan Polytechnic University, Jiaozuo, Henan, China; 4 University of Mining and Technology, School of Safety Engineering, Xuzhou, Jiangsu, China; 5 Shendong Coal Group Corporation Limited, erdos, Inner Mongolia, China; 6 National Key Laboratory of Earth System Numerical Modeling and Application, College of Earth and Planetary Sciences, University of Chinese Academy of Sciences, Beijing, China; UNSW: University of New South Wales, AUSTRALIA

## Abstract

To investigate the permeability enhancement effect of microorganisms (Methanogens) on anthracite and their impact on coal strength, static fracture tests were conducted on semi-circular SCB specimens with prefabricated cracks of varying angles after immersion in neutral and microbial solutions. The fracture process was monitored using digital image correlation DIC and VIC-2D techniques. The effects of different solutions and prefabricated angle cracks on the strength, deformation characteristics and fracture toughness of anthracite were analyzed. Simultaneously, the influence of the displacement field at the prefabricated crack tip of the coal sample during the failure process and the evolution characteristics of the strain field of the coal sample at different stages of the process were analyzed. The test results show that the composite specimens with different slit angles and different solution environments have significant differences in the loading process. The samples mainly experience tensile failure, with shear failure as a supplement. Fracture toughness decreased as the angle between the crack and loading direction diminished. In the microbial-solution environment, the ability of the samples to resist crack propagation is further reduced. Tensile deformation initiation occurred at 57.97 ~ 86.7% P_max_ for neutral solutions and 36.36 ~ 60.52% P_max_ for microbial solutions. Microbial solutions induced earlier crack tip tensile deformation, promoting tensile failure and extending the fracture process zone (FPZ) length.

## 1. Introduction

In China, coalbed methane (CBM) is a vital energy resource essential for energy security, optimizing the energy mix, and reducing pollution. However, China’s high-rank CBM reservoirs exhibit low porosity, low permeability, high adsorption, and high ground stress due to complex geology and burial conditions; well-developed micropores are often partially mineral-filled, further reducing permeability and pore-fracture connectivity. These limitations severely hinder extraction efficiency via direct drilling and horizontal wells. Hydraulic fracturing is therefore widely adopted to enhance permeability by creating interconnected fracture networks, significantly improving CBM recovery through high-pressure fluid injection into the coal seam. Using microbial solution as the fracturing fluid not only enhances seam permeability but also increases the coal matrix surface area, creating favorable conditions for microbial colonization and promoting CBM generation.

The biotransformation of coal into methane has become an important research topic in recent years [1,2]. Guo et al.‘s biomethane simulation experiments demonstrated significant post-metabolism changes in coal fractures: pore volume and connectivity increased, pore structure improved, and specific surface area decreased [3,4]. Lin used quantitative characteristic to investigate the spectrum of pore structure in medium and low rank coal. Low rank coal has more heterogeneity, more complicated structure, and more developed pores than medium rank coal [5]. Xia et al. studied the interaction between pore differences and biomethane production of different coal rank coals. A large amount of methane adsorbed in pores is desorbed, which is beneficial to the increase of coalbed methane production [6]. Pan et al. analyzed the micro-pores and cracks of coal by scanning electron microscopy and fractal theory [7–9]. Thommes et al. quantitatively analyzed the variation of pore structure of bituminous coal before and after treatment with Firmicutes, focusing on the change of pore volume in different pore size ranges of coal samples [10–12]. Although the literature on microbial enhanced coal permeability has been recorded, previous studies have used broken coal samples for testing. The fracture mechanics of intact block coal samples, especially the effect of microbial erosion on the fracture toughness and crack orientation of coal samples, has not been fully studied.

Among them, Nejati et al. carried out 124 pure mode I conducted fracture toughness tests on two different kinds of anisotropic rocks, and the weaker foliation or bedding plane is typically where the cracks in anisotropic rocks bend [13]. Dutler et al. studied the relationship between fracture toughness, tensile strength and fracture process bands in anisotropic rocks [14]. Wang et al. combined dynamic and static with loading tests on saturated samples, saturated water significantly impacted the strength and deformation of coal samples [15–17]. Wei et al. studied notch types and loading methods. Brazilian radial compression and three-point bending have a significant effect on fracture toughness measurement [18,19]. Yang et al. used the three-point bending test method to carry out the mode I fracture test on the pre-split semi-circular coal sample, and used the non-contact full-field strain measurement system to monitor and analyze the strain field evolution law and fracture process during the deformation and failure process of the coal sample [20]. The strain behavior of SCB specimens compacted under varying loading levels was examined by Zhang et al. Lastly, DIC performed a quantitative analysis of the FPZ and crack tip opening displacement (CTOD) [21–24]. Some studies took into account the influence of bedding direction in the experiment, yet overlooked the impact of the randomness of crack generation on the complex changes in the relative position of the two. As a result, there remains a substantial gap between the existing research findings and engineering applications.

Therefore, by combining the real-time DIC displacement tracking with the fracture toughness measurement of SCB specimens at different crack angles, we show the influence of different crack angles on the fracture mechanics of metamorphic coal samples, and reveal how microbial erosion affects crack formation.In this paper, fracture tests of coal samples with different angles of prefabricated cracks and different solution environments were conducted. The fracture loads of semi-circular bend (SCB) specimens were measured to obtain critical mechanical parameters, including mode I fracture toughness. It was revealed how coal samples were deformed and how they failed as cracks spread. Scanning electron microscopy (SEM) and high-speed digital image correlation (DIC) were used in an integrated analysis to shed light on the evolution of mesoscale damage under progressive loading. The effect of microbial degradation on the mineral phase composition of coal was systematically analyzed by X-ray diffraction (XRD) test, and the change of diffraction peak intensity of characteristic minerals was tracked. At the same time, combined with the statistical analysis of crack offset, the law of stress transfer path of coal under the action of microorganisms is revealed from the macro scale. These findings offer experimental insights into the mesoscopic mechanisms of mining-induced disasters and propose cost-control strategies for hydraulic fracturing operations. The above-mentioned research results can provide practical technical guidance for engineering practices such as controlling the development of the coal-seam network and enhancing the efficiency of coalbed methane extraction.

## 2. Sample preparation and test methods

### 2.1. Specimen preparation

**Fig 1:**
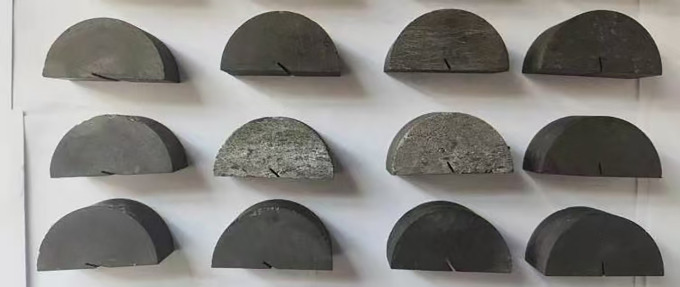
Part of the sample diagram.

The coal sample used in the experiment was anthracite. The standard sample for each group of tests was processed from a large, intact coal sample. First, a drilling rig with a diameter of 50 mm was used to core the coal sample, which was then processed into a cylindrical sample. The cylindrical coal sample was then sliced into a disc-shaped sample that measured 25 mm in thickness and 50 mm in diameter. Along the cutting line in the middle of the disk, it was divided into two semi-discs. Then, p repared fractures measuring 5 mm in length and 1 mm in width were cut from the center of the semi-circular disc perpendicular to the diameter direction using a high-speed rotating diamond wire with a thickness of 0.1 mm.To guarantee the sample’s experimental precision, the produced semi-circular disc sample was lastly polished using a grinding machine, as seen in Fig 1.

### 2.2. Solution immersion

**Fig 2:**
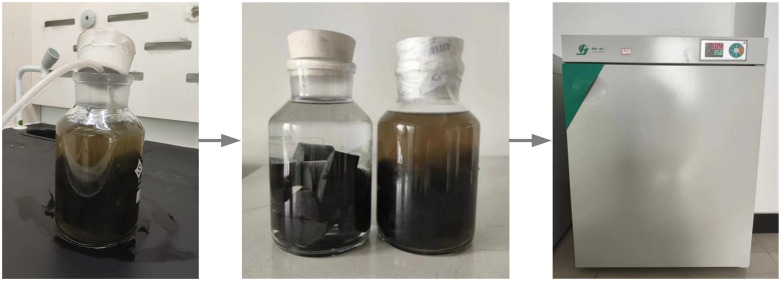
Immersion device and thermostat box.

In this experiment, neutral solution and microbial solution were utilized. The neutral solution was a distilled-water solution, and the microbial solution was a culture solution containing methanogenic bacteria. Before soaking the coal sample, its mass, height, and thickness were first measured. After the measurement, the sample was placed in a chemical container, and the required solution was poured into it. Moreover, methanogenic bacteria are anaerobic bacteria. In an anaerobic environment at a temperature of 35°C, the strain is most active. First, the container should be emptied oxygen, then sealed with a rubber plug, and placed in a 35°C incubator. The soaking time was 60 days, as depicted in Fig 2.

### 2.3. Test scheme

**Fig 3:**
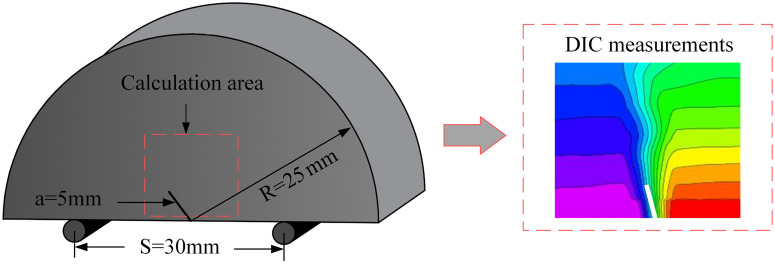
Sample size and span size diagram.

**Fig 4:**
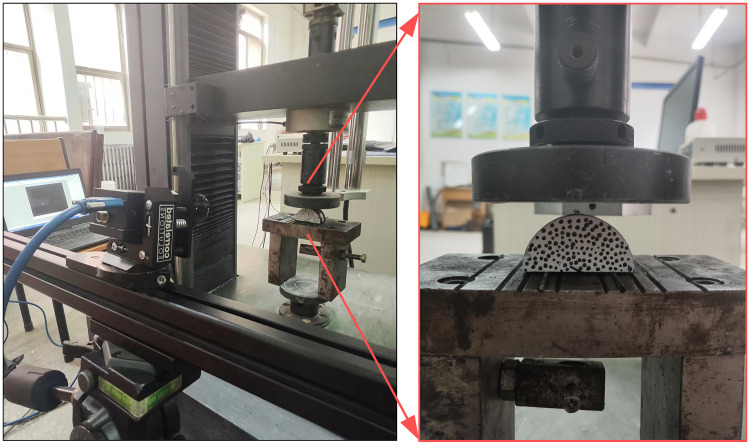
Test device and process.

(1) This test employed RMT-150C rock mechanics test system and image acquisition system. Universal testing machine is often used to test the tensile, bending, compression, shear and other mechanical properties of materials. The test machine is mainly composed of a motor drive device and an integrated, digital closed-loop control device. The load loading method selects displacement control. The image acquisition system is a measurement system based on the principle of digital image correlation. To determine the surface feature changes of the measured object, an industrial camera captures the image and takes a comparison of the specimen’s surface features before and after deformation. Then, the corresponding coordinate changes of each pixel of the image are obtained by digital image correlation algorithm, so as to calculate the displacement field, strain field and deformation field data in the process, as shown in Fig 3.
(2) Before the experiment, the surface of the sample was dried, and matte white paint was quickly sprayed onto the sample’s surface to create a white background with speckles. After the white paint on the sample’s surface had dried, the dot-coating process was carried out. The three-point bending test device was fixed on the upper and lower pressure heads of the testing machine to ensure that the three cylindrical rollers were parallel. The sample was symmetrically placed on the 5-mm-diameter cylindrical roller, and the prefabricated crack was aligned with the upper pressure head. The round roller was in a line, as shown in Fig 4.
(3) The universal testing machine adopted a displacement-controlled loading mode, with a loading rate of 0.02 mm/min. At the start of the test, two sets of systems were manually triggered simultaneously to ensure the synchronization of the recording time. Images were taken with a high-speed camera. The picture interval was 500μs, and the frame rate was set at 2 frames per second. Once the coal sample was damaged, the recording was stopped, and the collected data was saved for further analysis.

## 3. Reaction mechanism and microscopic pore evolution analysis

### 3.1. Bacteria source and reaction mechanism

The source of bacteria used in this experiment was taken from the deep mine water of Guhanshan Mine, which was rich in methanogens adapted to anaerobic environment. In the long-term geological evolution process, this kind of bacteria has formed an efficient degradation ability of organic matter in coal matrix, especially for the hydrolysis and fermentation of complex organic matter in anthracite. The screening of flora is based on its biological anti-reflection potential for coal-changing the pore structure of coal through metabolism, thereby affecting its mechanical properties. After collection, the bacteria liquid was sealed quickly and stored at low temperature to maintain the activity of the bacteria group. The composition of the enrichment medium was designed to simulate the mine water environment and optimize the methanogenic efficiency: sodium formate and sodium acetate were used as direct carbon sources to promote the synergistic metabolism of acetogenic bacteria and methanogenic bacteria; NH_4_Cl provided the nitrogen source, and MgC1_2_·6H_2_O and K_2_HPO_4_ regulated the ion balance. Na_2_S and L-cysteine hydrochloride were used to maintain anaerobic conditions and reduce redox potential. The trace element solution and vitamin solution ensure the essential nutrients for the growth of the flora. The optimum pH value of the experiment was around 7.0.When the pH was lower than 6.0 or higher than 8.0, its growth and CH4 yield would be significantly affected.

**Fig 5:**
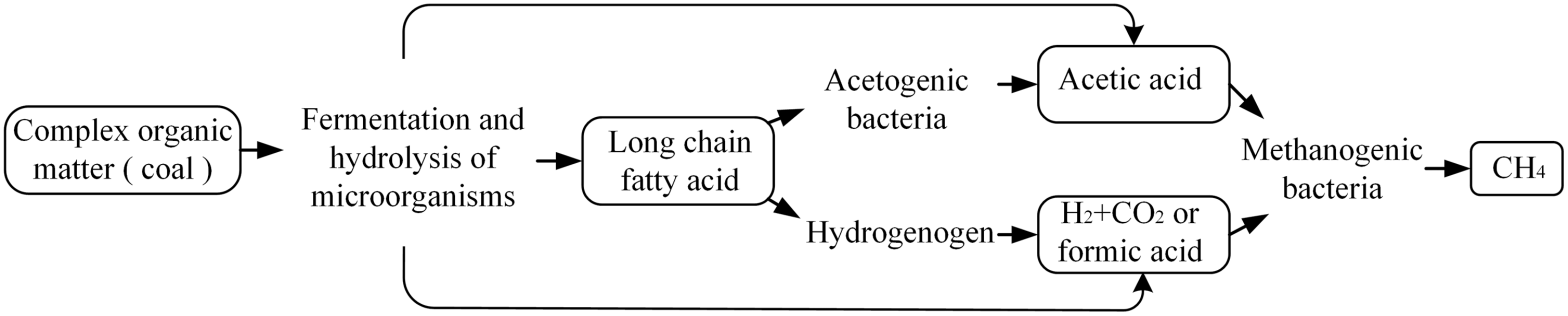
Microbial four-stage chain reaction mechanism.

**Fig 6:**
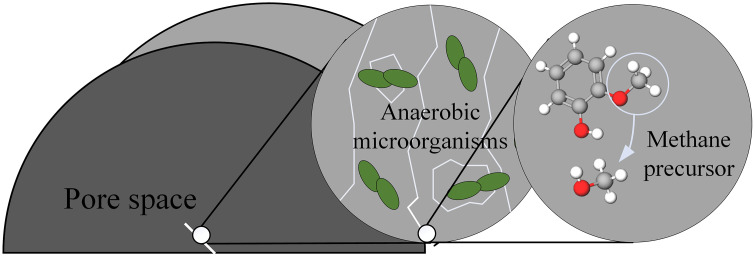
Microbial erosion diagram.

The formation of biomethane involves a four-stage chain reaction (hydrolysis, acidification, acetic acid production, and methanogenesis), as shown in Fig 5. The core is the stepwise degradation of organic matter in coal by microorganisms. In the hydrolysis stage, the fermentation bacteria secrete extracellular enzymes to decompose high-molecular polymers (such as lignin and humic acid) in coal into soluble monosaccharides and short-chain fatty acids. In the acidification stage, the hydrogen-producing acetogenic bacteria further converted the intermediates into acetic acid, H_2_O and CO_2_.Finally, hydrogenotrophic and acetic acidotrophic methanogens use H_2_/CO_2_ and acetic acid to produce CH_4_. In addition, the process of microorganisms using coal to produce methane precursor molecules is shown in Fig 6. Anaerobic microorganisms produce enzymes in the pores between coals, which destroy the macromolecular structure of coal by enzymatic reaction. The enzyme decomposes methoxy to produce methane precursor molecules, functional group shedding, aromatic ring breaking and pore connectivity enhancement.

**Fig 7:**
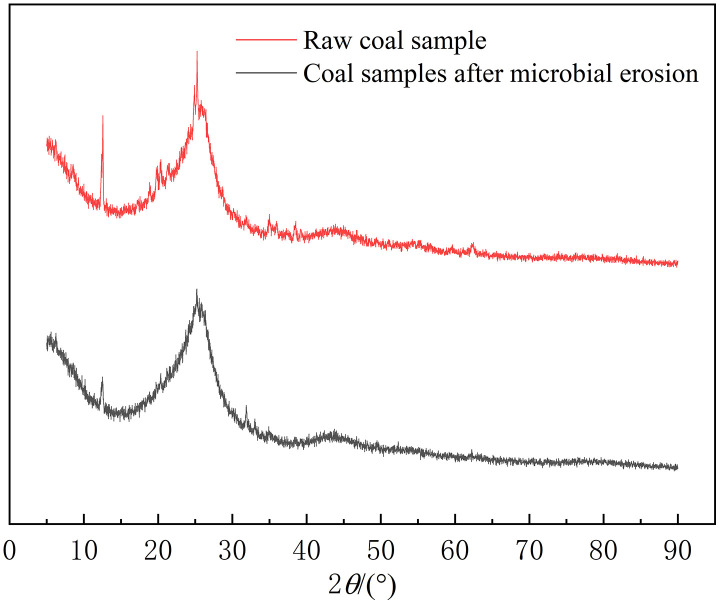
XRD patterns of coal samples before and after microbial erosion.

In order to explore the mineral changes caused by microorganisms, XRD test was used for composition analysis. The XRD pattern of coal samples was shown in Fig 7. The experimental results were analyzed by software MDI Jade, and the composition changes before and after coal samples were obtained. Among them, the content of Al₂SiO₅(OH)₄ in kaolinite decreases, which is clearly related to the erosion of microorganisms. In the process of anaerobic metabolism, although the high-efficiency methanogens degrade organic matter in coal, such as aromatic compounds and long-chain alkanes, as the main energy source, their metabolic activities can indirectly destroy the structure of kaolinite, weaken the cementation of clay minerals, and make cracks more likely to expand along the weakened bedding.

### 3.2. Fracture micromorphology characteristic

**Fig 8:**
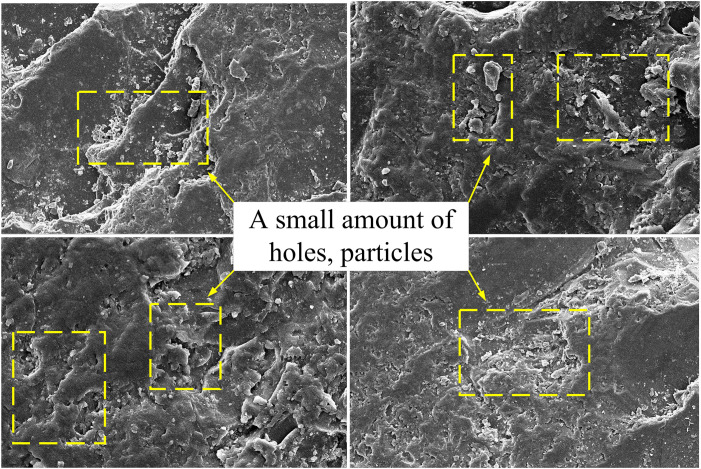
Results of scanning electron microscopy in neutral solution.

**Fig 9:**
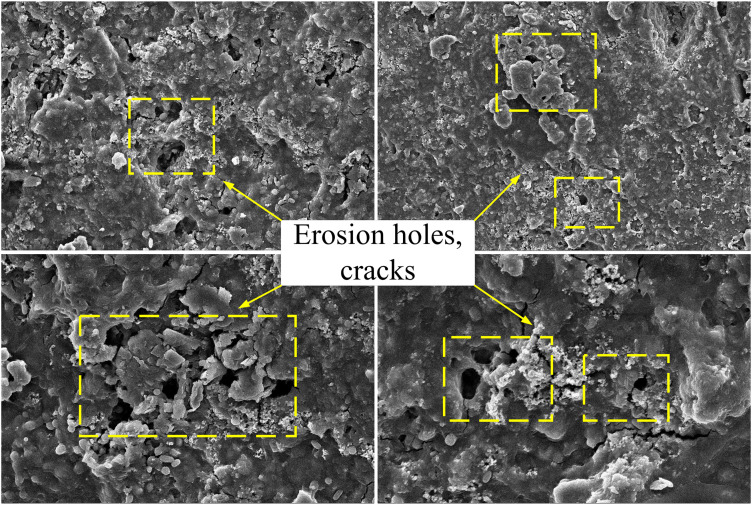
Results of scanning electron microscopy in microbial solution.

The macroscopic failure characteristics of coal samples are closely related to the internal microstructure. According to the reaction mechanism between microorganisms and coal samples, the characteristics of microscopic fracture surface can be studied, and the deformation details of crack propagation can be obtained. The coal samples under neutral solution and microbial solution were observed by scanning electron microscopy magnified by 5000 times, as shown in Figs 8 and 9.

The microstructural characteristics of the fracture surface of the coal sample under neutral-solution conditions were as follows: In the scanning area of the coal sample, the micro-fracture pores were relatively flat, showing no distinct pores or fissures, and there were a larger number of scattered crystal particles. The fracture surface was rough and three-dimensional, presenting a broken-block structure. Under microbial-solution conditions, the coal sample’s fracture surface exhibited the following microstructural features: The coal sample’s surface was uneven, with numerous small crystal fragments adhered to it. A large number of pores were developed, and the pores varied in size. Moreover, a large number of cracks were developed.

The presence of pores and the density of pore development indicated that the coal had strong gas-storage capacity. Cracks were relatively well-developed, and the connectivity between pores and cracks was favorable. The presence of a large number of pores reduced the mechanical strength of coal, which was also one of the reasons for the easy breakage of microbial-treated coal samples. This process significantly reduced the mechanical strength of coal. As shown in Table 1, the average fracture toughness of coal samples decreased by approximately 18% after microbial soaking, and as shown in Table 2, the length of the crack-propagation process zone increased. This indicates that microbial activity weakened the ability of coal to resist crack propagation. Furthermore, the acidic environment generated by microbial metabolites might have accelerated the dissolution of the coal matrix, thereby promoting the development of micro-fractures and accelerating tensile failure at a macroscopic scale. The interaction mechanism between the microbial flora and coal offered a microscopic explanation for the deterioration of the mechanical properties and the transformation of the fracture mode of the coal samples in this study (2013).

**Table 1:** Physical parameters.

Sample number	Crack angle/°	Peak load/N	Fracture toughness/Mpa.m1/2	Mean peak load/Mpa.m1/2	Mean value of fracture toughness/Mpa.m1/2
**B-1–1**	30°	694.700	0.223	694.100	0.223
**B-2–1**		699.348	0.225		
**B-3–1**		688.268	0.221		
**B-1–2**	45°	630.788	0.203	767.300	0.247
**B-2–2**		486.312	0.156		
**B-3–2**		1185.396	0.381		
**B-1–3**	60°	613.726	0.197	581.000	0.186
**B-2–3**		430.446	0.138		
**B-3–3**		698.828	0.224		
**B-1–4**	75°	675.206	0.217	594.670	0.191
**B-2–4**		685.280	0.220		
**B-3–4**		423.534	0.136		
**C-1–1**	30°	432.78	0.139	613.000	0.197
**C-2–1**		794.854	0.255		
**C-3–1**		611.366	0.196		
**C-1–2**	45°	480.920	0.154	601.138	0.193
**C-2–2**		517.324	0.166		
**C-3–2**		805.170	0.259		
**C-1–3**	60°	346.550	0.111	485.000	0.156
**C-2–3**		573.880	0.184		
**C-3–3**		534.570	0.172		
**C-1–4**	75°	579.184	0.186	537.070	0.173
**C-2–4**		346.816	0.111		
**C-3–4**		685.226	0.220		

**Table 2:** Maximum offset statistics.

Solution environment	30°maximum offset/mm	45°maximum offset/mm	60°maximum offset/mm	75°maximum offset/mm
**Neutral solution**	4.58	3.33	7.58	1.67
	1.78	10.11	5.83	2.51
	13.75	5.79	5.10	2.34
**Microbial solution**	5.10	4.55	3.17	2.92
	7.34	3.78	4.43	2.88
	6.15	5.37	2.35	1.74

### 3.3. Variation characteristics of pore distribution

The attachment and activity of methanogens (0.5 ~ 2.5μm in diameter) are strictly limited by the pore size. According to the International Union of Pure and Applied Chemistry (IUPAC) [10], micropores are defined as those with a diameter less than 50nm, and these micropores serve as the primary sites for microbial activity, being sufficiently sized to accommodate bacterial metabolic processes. Microorganisms significantly improve pore connectivity by degrading coal matrix in macropores. At the same time, the gas produced by metabolism will further expand pore throat channels and form a penetrating fracture network.

After soaking in neutral solution, the pore structure of coal samples was dominated by isolated micropores, with smooth pore walls and poor connectivity (porosity<5%).In summary, gas production during coal fermentation to methane is influenced not only by the bioavailability of coal but also by the degree of contact between the coal and microorganisms. These two factors are complementary. Biomethane metabolism changes the pore structure of coal by biological enzymes acting on its macromolecular structure, causing functional groups and side chains to fall off, benzene rings to open, oxygen content to increase, and the degree of crystallization of coal to decrease [25,26]. The experimental data obtained by Xia indicated that the microbial action significantly modified the pore distribution pattern [27]. In the neutral environment, the pore size was concentrated in the range of 0.1 ~ 1μm, accounting for 68%. In contrast, in the microbial environment, the proportion of pores with a size of 1 ~ 10μm increased to 52%, and local transfixion cracks larger than 10μm emerged. This was consistent with the high dispersion of the FPZ length data and the FPZ range of 10.33 mm in the 30° microbial environment, suggesting that the alteration of the local pore network would preferentially lead to crack deflection. Moreover, long-term soaking might have led to the excessive development of the pore structure and a reduction in the strength of the coal body. This phenomenon of coordinated development of multi-scale pores directly resulted in a decrease in the specific surface area of the coal body [28]. Such a decrease was conducive to the desorption and migration of coalbed methane and held important potential significance for the development of coalbed methane.

## 4. Experiment results and analysis

### 4.1. Load-displacement curve

**Fig 10:**
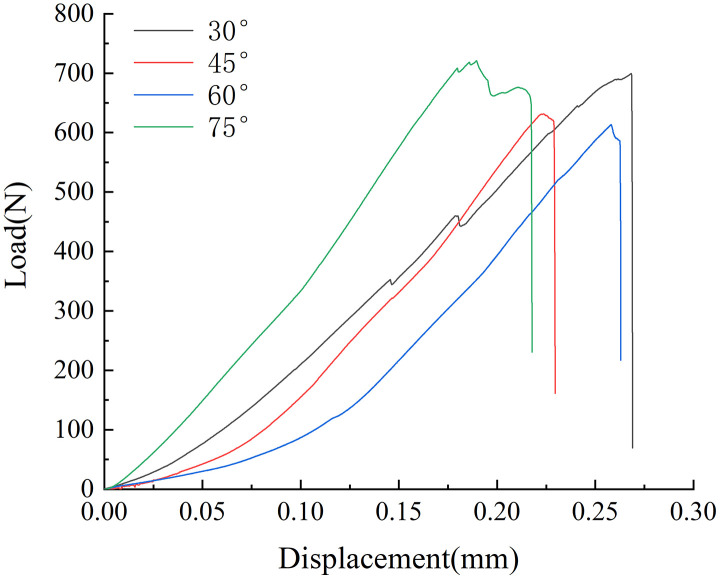
Load displacement curve in neutral solution.

**Fig 11:**
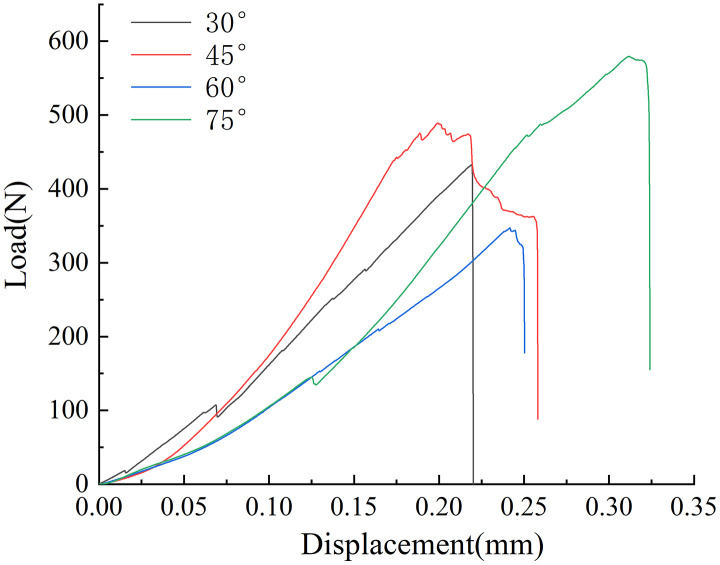
Load displacement curve in microbial solution.

Figs 10 and 11 shows the load-displacement curves of several samples. The mechanical properties of the samples were closely associated with the solution environment and the angle of the prefabricated crack. The load-displacement curve could be roughly categorized into three stages: compaction, elasticity, and crack initiation and propagation. When the specimen attained the fracture load, the curve dropped vertically and, upon a crisp sound, the specimen broke, demonstrating brittle characteristics.

Compaction stage: The load increased slowly, and the displacement increment was substantial, reflecting the process of microcrack closure and pore compaction within the sample. In the microbial environment, the slope of this stage was slightly smaller than that in the neutral environment, suggesting that microbial activity exacerbated the initial damage to the coal. Elastic stage: The relationship between the load and displacement was linear, and the coal body mainly underwent elastic deformation. The elastic modulus of the sample in the neutral solution (with an average value of 3.2 GPa) was significantly higher than that of the sample in the microbial environment (with an average value of 2.3 GPa). This result validated the weakening effect of microbial degradation on the stiffness of the coal. Initiation and propagation stage: The load dropped sharply after reaching its peak value. Accompanied by the sound of a crisp fracture, the crack propagated rapidly from the prefabricated tip until the specimen failed. In the microbial environment, the curve dropped more steeply at this stage, suggesting that the resistance to crack propagation was further decreased. The average fracture loads of the coal samples in the neutral solution with different pre-crack angles were 693.9N, 767.3N, 581N, and 594N, respectively. The average fracture loads of the coal samples in the microbial solution were 613N, 600N, 485N, and 537N, respectively. The average fracture load of all specimens in the microbial environment was approximately 18 ~ 23% lower than that in the neutral environment. Moreover, as the angle of the prefabricated crack increased, the average fracture load exhibited a decreasing trend. The sample with a pre-crack angle of 75°had the smallest decrease, only 9.6%, suggesting that microorganisms had a more pronounced effect on low-angle tensile failure. In conclusion, this phenomenon might be attributed to the development of the pore structure in the coal samples under the microbial environment. The activity of microorganisms reduces the strength of coal. Additionally, different prefabricated cracks led to variations in the mechanical strength of the samples. The fracturing strength of 60 ° coal sample is higher than that of 75 ° coal sample, which is the result of the combination of crack propagation path and internal bedding plane matching and coal heterogeneity. As a typical heterogeneous material, the coal body contains a large number of primary microcracks and mineral bodies, and its distribution is random. If the coal body such as the primary crack and the prefabricated crack intersect at a small angle, the stress of crack propagation will be significantly reduced, resulting in more significant initial damage of the 60 ° specimen.

### 4.2. The variation law of effective fracture toughness

Fracture toughness (K_IC_) is the ability of materials to prevent the unstable propagation of cracks under the condition of crack propagation. It serves as a crucial indicator for evaluating the ability of a material to resist crack propagation. Based on the fracture load of the sample acquired from the test and in accordance with the ISRM standard, the calculation formulas for determining the mode-I fracture toughness of coal samples under stress are presented in equations (1) and (2).


$$K_{IC}=YP_{max}\sqrt{\pi a}/\text{2}RB$$ (1)

$$\text{Y}=-\text{1}.\text{297}+\text{9}.\text{516}a_{\alpha }-\left[\text{0}.\text{47}+\text{16}.\text{457}a_{\alpha }\right]\beta +[\text{1}.\text{071}+\text{34}.\text{401}a_{\alpha }]\beta^{\text{2}}$$ (2)

In this formula, Y represents the dimensionless strength factor, P_max_ represents the specimen’s peak load, a represents the length of the prefabricated crack, R represents the specimen’s radius, B represents the specimen’s thickness, a_α_ represents the ratio of the diameter of the specimen D to the span of the two supporting points, and β represents the ratio of the prefabricated crack a to the radius of the specimen R[29], where a_α_ = 0.6, β = 0.2.

Table 1 summarized the peak-load and fracture-toughness parameters under different solution environments. The analysis revealed that in the neutral-solution environment, the fracture toughness fluctuated as the prefabricated crack angle increased. When the prefabricated crack angle changed from 30° to 75°, the average value of the fracture toughness ranged from 0.223 to 0.191 MPa·m^1/2^, and the fracture toughness of the coal sample gradually decreased. This may be explained by the prefabricated crack’s effect on the tensile stress, which facilitates the crack initiation from the crack tip and reduces the difference. Likewise, the fracture toughness of coal showed a declining tendency in the microbial-solution environment. Because the heterogeneity of coal, the test findings showed that the average fracture of 60° sample toughness was lower than the 75° under both neutral and microbial conditions. In summary, the fracture mode and toughness of the sample are significantly influenced by the solution environment and the prefabricated crack angle.

**Fig 12:**
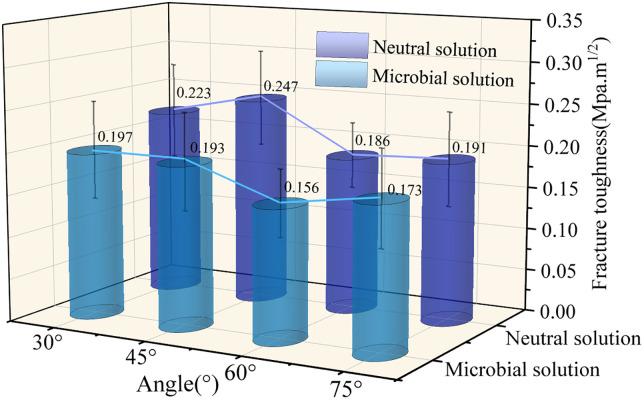
Variation characteristics of fracture toughness of coal samples with solution environment.

As can be observed from Fig 12, the ability of coal samples to resist crack propagation in the neutral environment was higher than that in the microbial-solution environment. In other words, the average fracture toughness in the neutral-solution environment was higher than that in the microbial-solution environment. The average fracture toughness of coal samples indicated that the physical and mechanical properties of coal samples were degraded to varying degrees under microbial conditions.

### 4.3. Analysis of specimen failure mode

**Fig 13:**
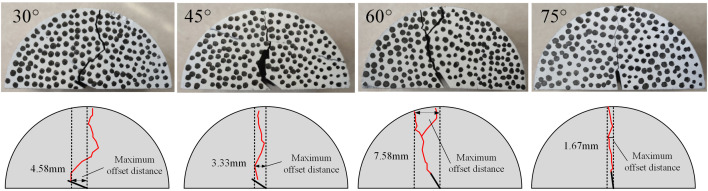
Typical fracture morphology of coal samples in neutral solution environment.

**Fig 14:**
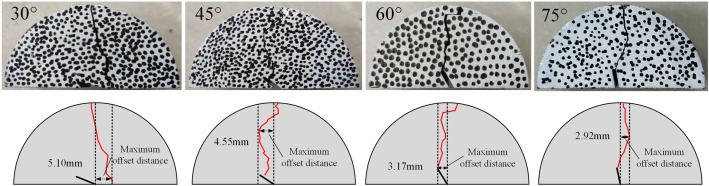
Typical fracture morphology of coal samples under microbial solution environment.

Figs 13 and 14 showed the typical fracture morphology of coal samples under different prefabricated crack inclination angles (30°,45°,60°,75°) in different solution environments (neutral solution environment and microbial solution environment). During the loading process, the main cracks of the coal samples all initiated from the prefabricated crack tip and propagated in the loading direction.

In the neutral-solution environment, the loading-failure mode of the 30° coal sample initiated from the prefabricated crack, yet the propagation path began to deviate to a certain degree. The failure modes of the 45°and 60° coal samples were still predominantly tensile failure. However, during the crack-propagation process, a small number of branches might emerge, and shear failure increased, resulting in the formation of a complex crack network. The crack-propagation direction of the 75° coal sample was essentially consistent with the direction of the prefabricated crack. The crack propagated more readily along the prefabricated crack direction, and the propagation path was relatively straight.

In the microbial-solution environment, the 30° coal sample cracked from the right-hand cylindrical support point, and its propagation path was relatively straight. The crack-propagation direction of the 45° coal sample deviated significantly from the prefabricated crack direction. Microorganisms might have accelerated the crack propagation and branching. The failure mode of the 60°coal sample was similar to that in the neutral-solution environment, and its expansion path was evidently curved. The expansion path of the 75° coal sample was relatively straight.

Among them, the angle between the prefabricated crack and the loading direction of the 30°coal sample was relatively large. Before and after soaking, the coal sample exhibited a case of cracking from the right-end cylindrical support point [29]. This discrepancy resulted from the prefabricated cracks were all slits on the left side, showing a significant difference from the loading-direction angle. During the loading process, the force-application point shifted to the right side, leading to cracking from the right-end cylindrical support point. The 45°, 60° and 75° coal samples were mainly damaged starting from the prefabricated cracks. The failure mode was mainly tensile failure, accompanied by shear failure. Under the combined effect of the solution environment and different opening angles, the fracture process of the coal samples was more complex, mainly in the form of tensile and shear deformations at the tip of the prefabricated crack. In the figures, the crack path in the microbial-solution environment was more complex than that in the neutral-solution environment. It is likely that the microorganisms had a certain impact on the physical properties of the coal sample, accelerating the crack’s expansion and branching [30].

Taking the central axis of the sample as the reference, the maximum offset distance of the crack propagation path relative to the central axis was measured, as illustrated in Figs 13 and 14. The prefabricated crack is located at the geometric center of the sample; in a uniform stress field, its ideal propagation path should align with the central axis. Therefore, the offset can reasonably characterize the degree of interference exerted by the prefabricated crack and microbial erosion on the crack propagation path.

**Fig 15:**
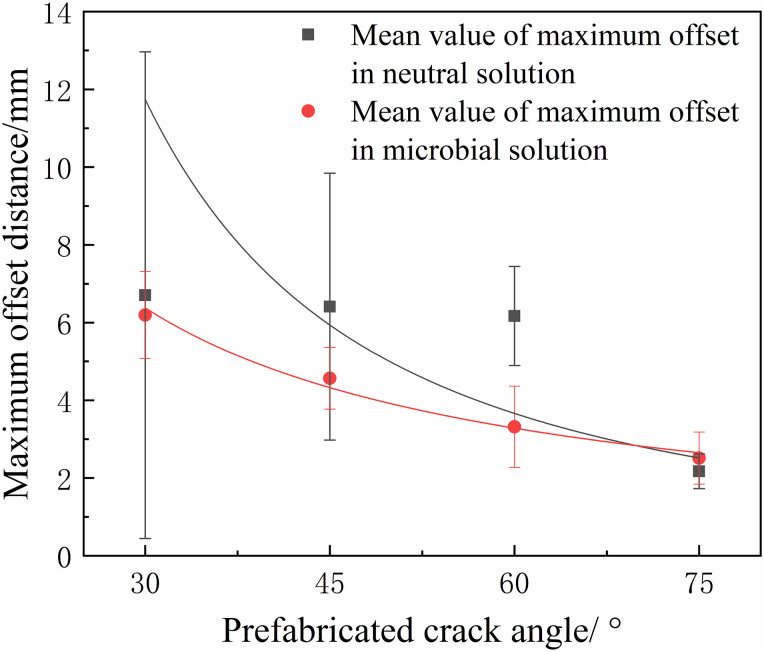
Fitting law of maximum offset under different solutions.

The offsets of all samples were measured, and their average values were computed, as shown in Table 2. According to the statistical results of the offsets, the crack offset of microbially immersed samples is smaller than that of samples in a neutral environment, as presented in Fig 15. Microorganisms degrade organic matter and dissolve soluble minerals through metabolism, thereby altering the homogeneity of the internal structure of the coal mass and the distribution of crack propagation resistance, which in turn affects the stability of the crack propagation path. In a microbial environment, the fracture toughness of coal decreases, accelerating crack propagation, shortening the “response time” to local heterogeneous structures, and making the crack less likely to be significantly deviated from the main direction by a single defect. These combined effects facilitate more stable propagation of cracks along the principal stress direction, ultimately resulting in a reduced offset.

Several studies have shown that the crack-development trajectory and its kind are highly complex when assessing the fracture behavior of specimens with a single crack under uniaxial compression. Owing to the coupling effect of the prefabricated crack and bedding, the shear crack does not invariably develop along the pre-cracking direction. Judging the fracture form merely based on the crack trajectory is inconclusive. Therefore, the fracture mechanism of the normal displacement of coal samples was investigated using digital image correlation technology. However, prior research has mostly focused on the displacement-distribution properties on both sides of the fracture at a particular point in time. The crack-initiation time and the crack-initiation mode can be more accurately reflected by the real-time displacement characteristics, which have not been thoroughly studied. Thus, it is essential to track the crack displacement in real time and investigate the specimen’s fracture-propagation mechanism at various loading angles.

## 5. Analysis of deformation field of coal sample

### 5.1. Basic principles of DIC

The fundamental principle of DIC involves tracking and matching the position of the same pixel in two speckle images captured before and after surface deformation of an object to obtain the displacement vector of that pixel. This enables the derivation of the variation law governing the full-field displacement and deformation fields on the specimen surface.

**Fig 16:**
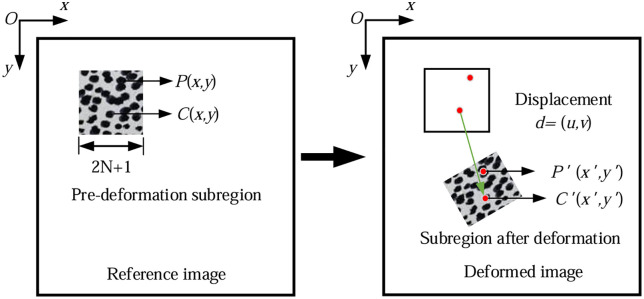
The principle of digital image correlation technology.

In this experiment, digital image correlation analysis was employed to observe full-field displacement and determine the crack tip position. As shown in the Fig 16, a correlation function is used to match the (2N + 1)×(2N + 1) sub-region in the reference image with the corresponding sub-region in the deformed image. By minimizing or maximizing the correlation coefficient, the position of the sub-region in the deformed image can be identified, and its displacement can be determined. The displacement of other pixels within the sub-region can also be calculated using the gradient of the displacement components.

### 5.2. Analysis of displacement field evolution of surface crack of coal sample

**Fig 17:**
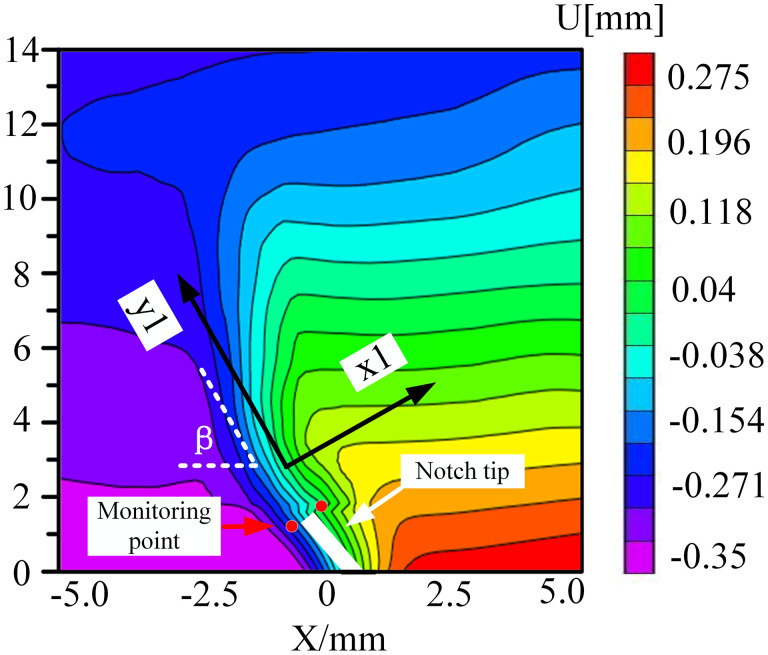
Displacement field X_1_OY_1_ coordinate.

The mode-I fracture specimen initiated cracking from the crack tip and then propagated in the loading direction. To more precisely collect the deformation of brittle coal samples, DIC technology was employed to track and record the deformation changes on the sample surface. To monitor the real-time variation of displacement information with time, The Zhao et al. approach was used to create a new local coordinate system. With the monitoring point as the coordinate origin, the x1 axis was perpendicular to the crack direction, and the y1 axis was parallel to the crack direction in Fig 17. Next, normal and tangential displacements (u1, v1) were created from all horizontal and vertical displacement components (u, v) [31].

Among them, the horizontal displacement field and vertical displacement field after coordinate transformation were shown in formulas (3) and (4).In summary, the relevant information of horizontal opening displacement and vertical slider displacement was obtained, and then the failure mode of coal sample fracture under different environments was understood.


$$u_{\text{1}}=\text{usin }\beta +\text{vcos }\beta$$ (3)

$$v_{\text{1}}=-\text{ucos}\beta +\text{vsin}\beta$$ (4)

where β is the angle at the monitoring point between the crack direction and the horizontal direction.

**Fig 18:**
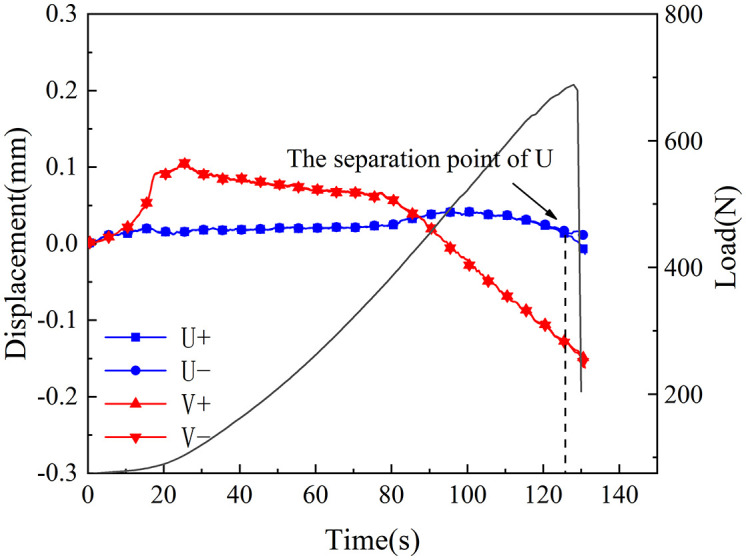
Displacement evolution law of crack tip of 30°coal sample in neutral solution.

**Fig 19:**
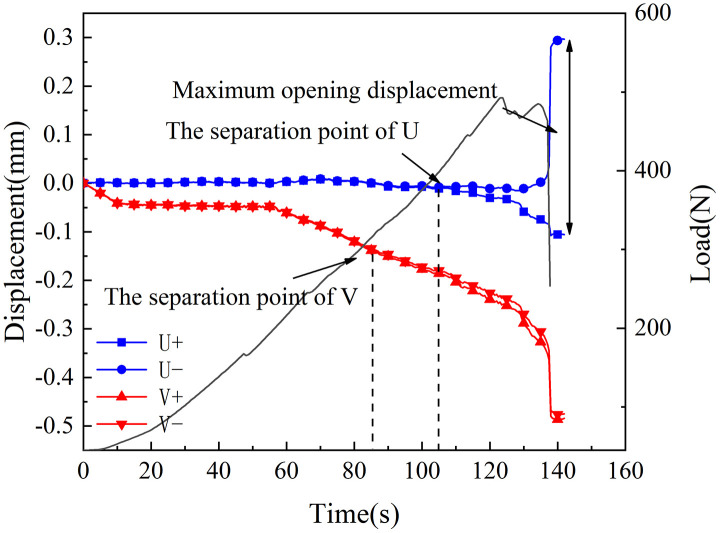
Displacement evolution law of crack tip of 45°coal sample in neutral solution.

**Fig 20:**
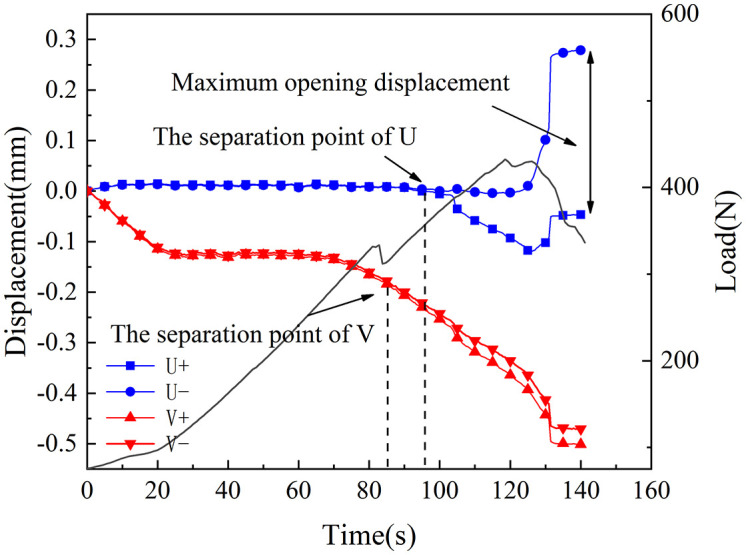
Displacement evolution law of crack tip of 60°coal sample in neutral solution.

**Fig 21:**
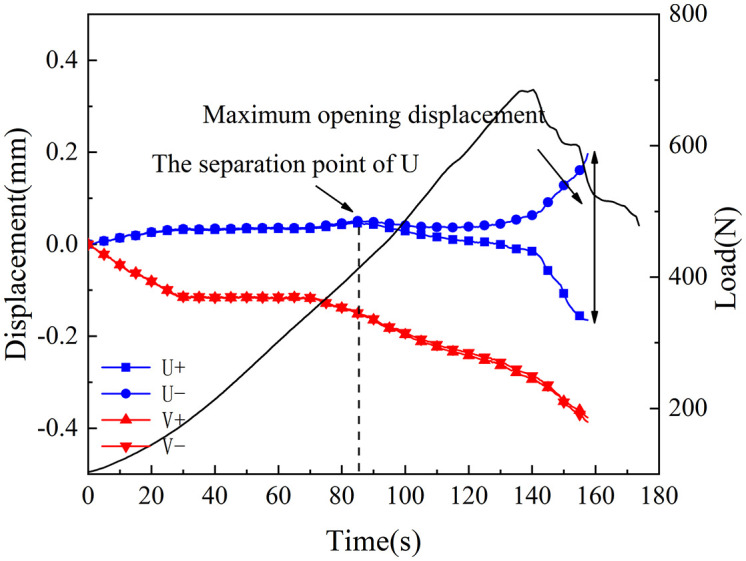
Displacement evolution law of crack tip of 70°coal sample in neutral solution.

**Fig 22:**
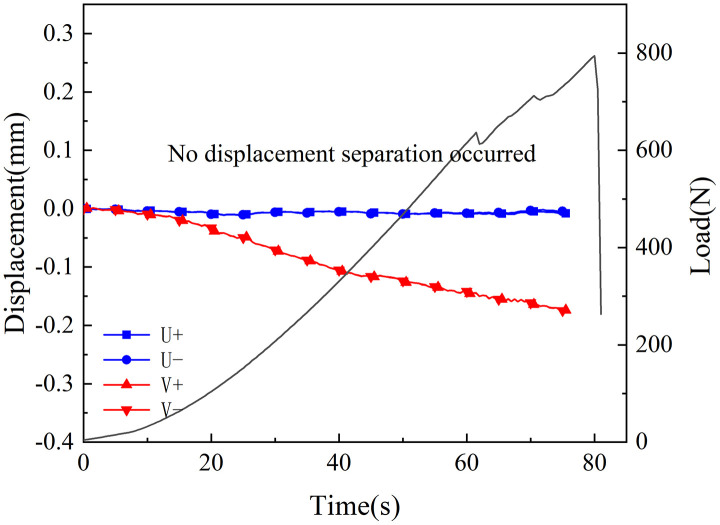
Displacement evolution law of crack tip of 30°coal sample in microbial solution.

**Fig 23:**
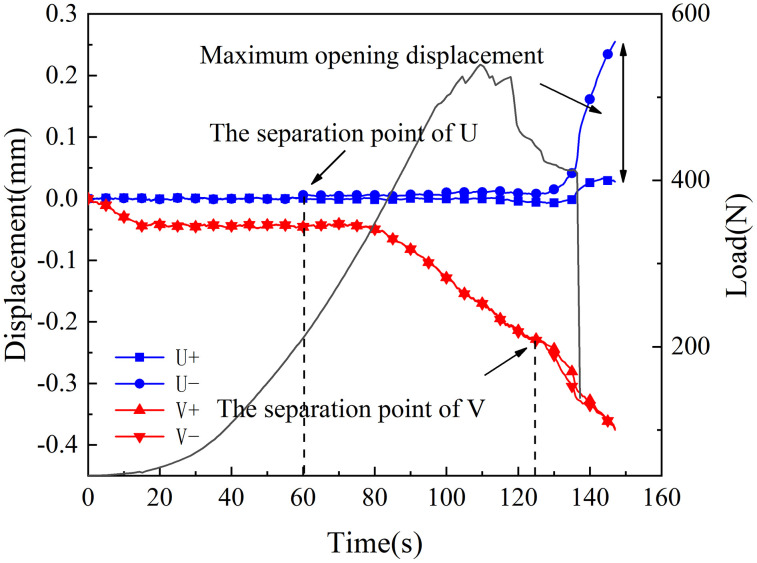
Displacement evolution law of crack tip of 45°coal sample in microbial solution.

**Fig 24:**
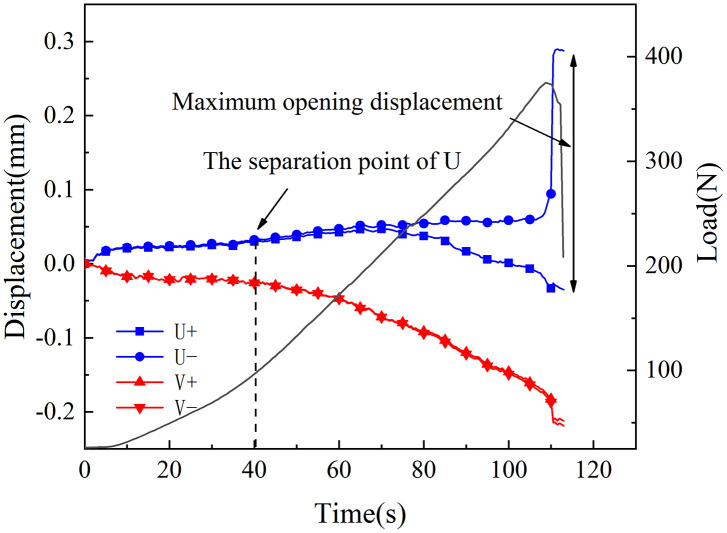
Displacement evolution law of crack tip of 60°coal sample in microbial solution.

**Fig 25:**
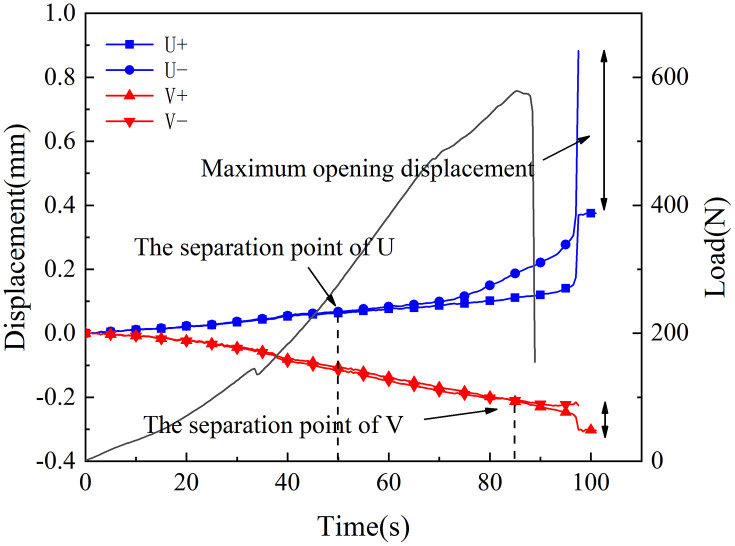
Displacement evolution law of crack tip of 70°coal sample in microbial solution.

Figs 18–25 illustrate the analysis of the evolution features of the monitoring points’ tangential and normal displacements throughout time. During the first phase of compaction, there was no separation and the monitoring point’s tangential and normal displacements were roughly zero. It was evident from this that the sample’s micro-cracks and pores were at the micro-crack compaction stage. In the later stage of compaction, as the load increased, the displacement deformation gradually augmented. The normal displacement underwent relative deformation along the positive and negative directions of the x1-axis and reached the maximum normal displacement at the peak moment, leading to tensile failure. The tangential displacement underwent relatively small deformation along the opposite direction of the y1-axis, culminating in shear failure [11].

The normal displacement generally tended to the positive direction of the x1-axis.This could be attributed to the uniformly-placed prefabricated crack inclination angle in the opposite direction of the x1-axis.During the vertical-compression process, the crack inclination angle caused the failure mode of the SCB specimen to change, making it tend to the positive direction of the x1-axis.The tangential displacement is generally negative and tends to be in the opposite direction of the y1 axis. During the entire pressurization process, the monitoring points of the sample always moved downwards. The asymmetry of the deformation on both sides of the sample reflected the inherent heterogeneity of the anthracite.

As can be inferred from Figs 18 and 19, the 30° specimen in the neutral-solution environment exhibited almost no tensile or shear failure. In contrast, for the 45° specimen, separation initiated from the horizontal displacement of the two monitoring points at t = 105s (86.7% of P_max_), at which point tensile failure occurred, with the maximum normal displacement reaching 3.8 × 10^−1^mm.Shear failure took place at t = 83s (66.89% of P_max_), and the relative shear displacement was negligible.

Overall, the prefabricated crack tip of anthracite experienced mainly tensile failure, with shear failure as a supplement. Based on all the samples, the initial range of the load level corresponding to tensile deformation in the neutral-solution environment was 57.97 ~ 86.7% of P_max_, while that in the microbial-solution environment was 36.36 ~ 60.52% of P_max_. The tensile deformation of the crack tip in the microbial-solution environment preceded that in the neutral-solution environment.

### 5.2. Study on the length change of fracture process zone

**Fig 26:**
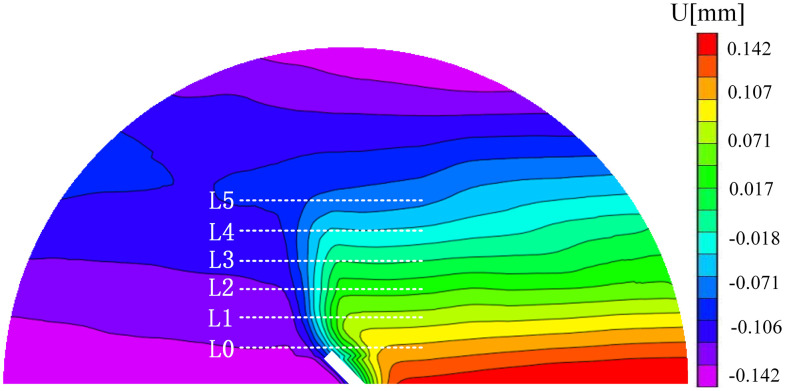
Horizontal survey line distance diagram.

**Fig 27:**
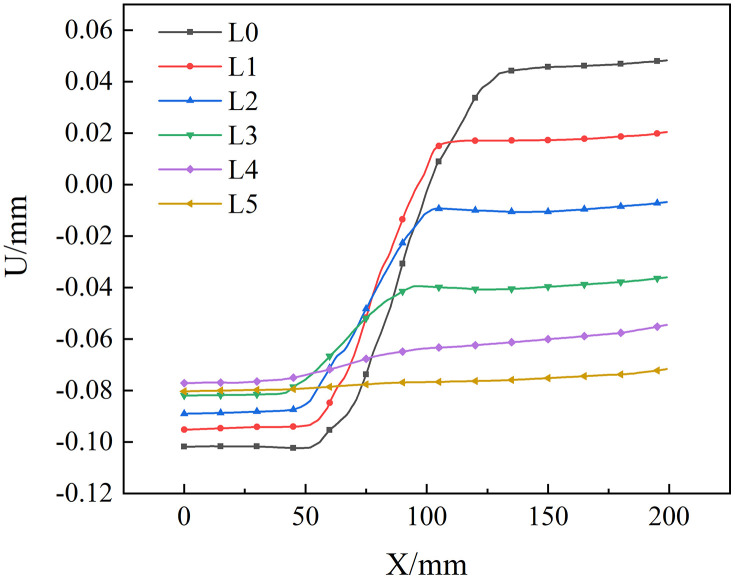
Displacement distribution of horizontal survey line.

Understanding the failure mode of the specimen greatly depends on the FPZ, which is the damage area at the specimen’s crack tip and releases the singularity. The Lin et al. approach is used to determine the length of the FPZ [5]. As shown in Fig 26, firstly, the first horizontal survey line at the tip of the prefabricated crack is L0, that is, y = 0 mm, and then the horizontal survey line is arranged every 2.5 mm in the monitoring area. A survey line is arranged until the survey line is horizontal, L0 ~ Ln, and the length of the FPZ is calculated. Taking sample B-2–2 as an example, the displacement distribution along the horizontal survey line was obtained. The analysis is shown in Fig 27.It can be seen that the horizontal displacements extracted from the L0 ~ L4 survey lines all have obvious jump phenomena. In addition to the mutation part, the rest of the displacement is approximately horizontal, and the displacement discontinuity occurs at the tip of the prefabricated crack on the surface. Until the measuring line L5, y = 12.5 mm, the horizontal displacement extracted by the horizontal measuring line does not jump, and the curve is relatively straight, indicating that the measuring line L5 is the boundary of the FPZ, so the length of the FPZ of the sample is 12.50 mm. The distance between the measuring lines of 2.5 mm may lead to insufficient measurement accuracy of the length of the fracture process zone. In order to obtain more accurate numerical results, the position of the boundary measuring line is further fine-tuned to make it more suitable for the length of the FPZ and calculated.

Based on the variation pattern of the FPZ length presented in Table 3, the data-discreteness analysis further revealed that the FPZ lengths of the 30° samples in the neutral-solution environment were 12.72 mm, 13.92 mm, and 5.15 mm, respectively, exhibiting a large span. This might suggest that the heterogeneity of the coal samples had a substantial impact on the results. Additionally, in the microbial-solution environment, the FPZ lengths at the same angle also exhibited significant differences. For instance, the FPZ lengths at 30° were 10.11 mm, 20.44 mm, and 12.31 mm, respectively. This reflected the coupling effect of coal heterogeneity and microbial erosion.

**Table 3:** Length of FPZ.

Sample number	Pre-crack angle/°	Fracture process zone length/mm	Sample number	Pre-crack angle/°	Fracture process zone length/mm
**B-1–1**	30°	12.72	**C-1–1**	30°	10.11
**B-2–1**		13.92	**C-2–1**		20.44
**B-3–1**		5.15	**C-3–1**		12.31
**B-1–2**	45°	12.85	**C-1–2**	45°	17.52
**B-2–2**		12.50	**C-2–2**		19.23
**B-3–2**		8.23	**C-3–2**		14.84
**B-1–3**	60°	16.45	**C-1–3**	60°	16.50
**B-2–3**		17.01	**C-2–3**		20.92
**B-3–3**		12.44	**C-3–3**		18.297
**B-1–4**	75°	14.00	**C-1–4**	75°	15.99
**B-2–4**		17.02	**C-2–4**		17.88
**B-3–4**		18.01	**C-3–4**		16.21

In comparison with the neutral-solution environment, microbial activity resulted in a general increase in the FPZ length of the low-angle fracture (30° ~ 60°) samples. This was achieved through the degradation of the coal’s organic matter and the enhancement of pore connectivity. For instance, the average FPZ lengths of the 30°, 45°, and 60° samples increased by approximately 34.7%, 56.4%, and 21.4%, respectively. This phenomenon was associated with the weakening of the coal’s strength and the facilitation of crack-tip tensile failure by microbial metabolism. The electron-microscope results revealed that the pores and cracks were significantly developed in the microbial environment. The displacement-field analysis indicated that the initial time of tensile deformation was 36 ~ 60% earlier than the time corresponding to the peak load in the neutral environment. However, the increase in the FPZ of high-angle cracks (75°) was limited (only 2.1%).This was mainly because its failure mode was constrained by the relationship between the crack dip angle and the loading direction. When the prefabricated crack angle was close to the loading direction, the stress distribution at the crack tip was dominated by the tensile component (mode I), and the shear stress (mode II) was significantly decreased, rendering shear failure difficult to initiate. The deterioration of the coal’s mechanical properties by microorganisms was mainly manifested in the reduction of tensile strength. In contrast, the weakening effect on shear strength was limited, and the influence of microorganisms was partially offset. As a result, the increase in the FPZ of the 75° high-angle cracks was small.

### 5.3. The change of displacement cloud diagram in FPZ

**Fig 28:**
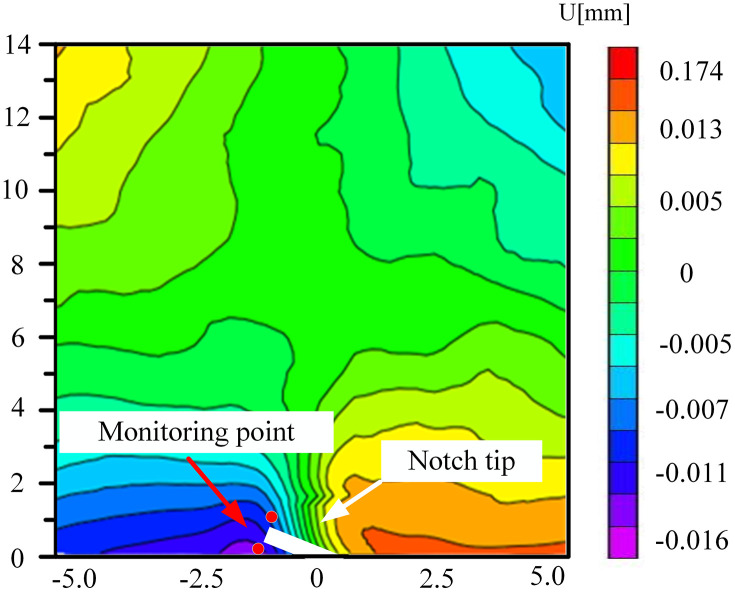
Horizontal displacement nephogram of 30°coal sample in neutral solution.

**Fig 29:**
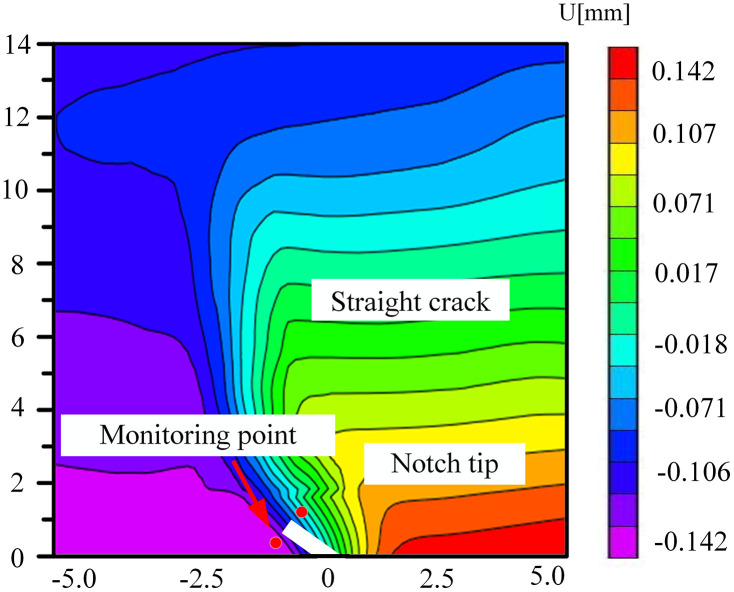
Horizontal displacement nephogram of 45°coal sample in neutral solution.

**Fig 30:**
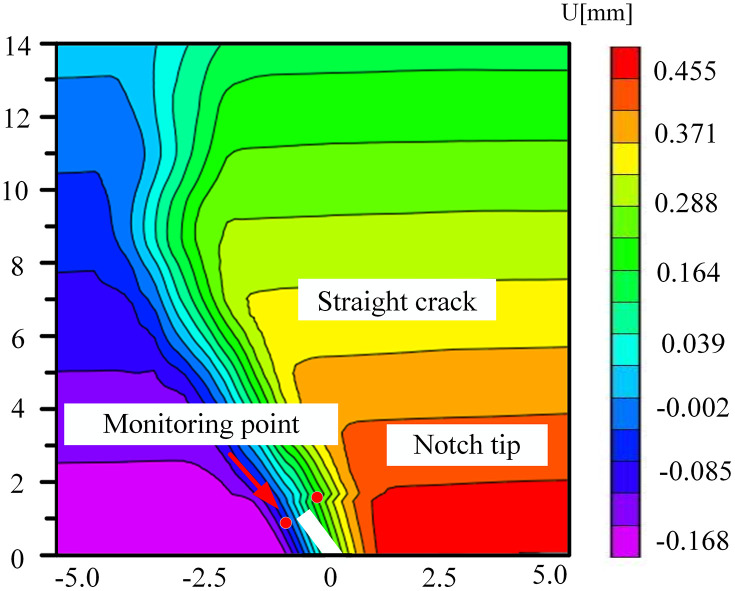
Horizontal displacement nephogram of 60°coal sample in neutral solution.

**Fig 31:**
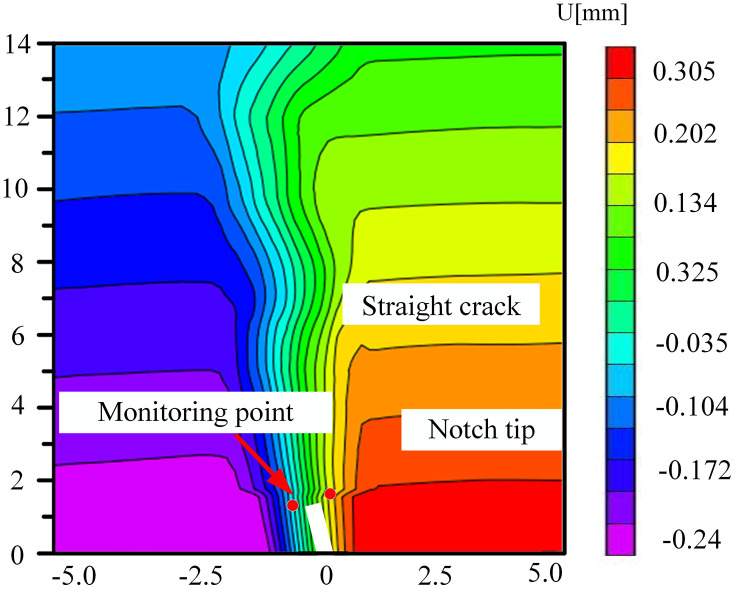
Horizontal displacement nephogram of 75°coal sample in neutral solution.

The coal samples immersed in the neutral solution mainly experienced physical infiltration, and the distribution of pores and fissures was relatively homogeneous. During the loading process, the variation of the displacement field was governed by the change in the angle of the prefabricated crack and the direction of the external load. Owing to the homogeneous mechanical properties within the coal body, the crack propagated steadily along the prefabricated crack tip or the direction of the maximum principal stress, as depicted in Figs 28–31. The displacement cloud diagram exhibited a continuous gradient change. The normal displacement component perpendicular to the crack direction was extremely small, and the tangential displacement parallel to the crack direction dominated. The cloud image displayed characteristics of vertical smoothing. The vertically-smooth displacement cloud diagram indicated that the crack-propagation path was controllable and was suitable for predicting the extension direction of the main crack in hydraulic fracturing. For instance, in low-angle fractured coal seams, the propagation of fractures along the maximum principal stress direction could be precisely controlled by adjusting the injection pressure of the fracturing fluid.

Microbial degradation created a local high-porosity region via erosion. These non-homogeneously distributed pores became stress-concentration points during the loading process, giving rise to local mutations in the displacement field in –. As depicted in Fig 34 for the 60° specimen, the displacement cloud diagram displayed an obvious ‘S’-type tortuous distribution along the direction of the crack extension, which corresponded to the through-crack formed by microbial activity. The crack bifurcated at this location and propagated along the low-strength region, leading to the cloud diagram exhibiting multi-peak tortuous characteristics. The tortuous displacement cloud diagram revealed the potential for the formation of a complex fracture network. Moreover, the multi-stage bifurcated cracks could significantly augment the surface area of the gas-seepage channel.

**Fig 32:**
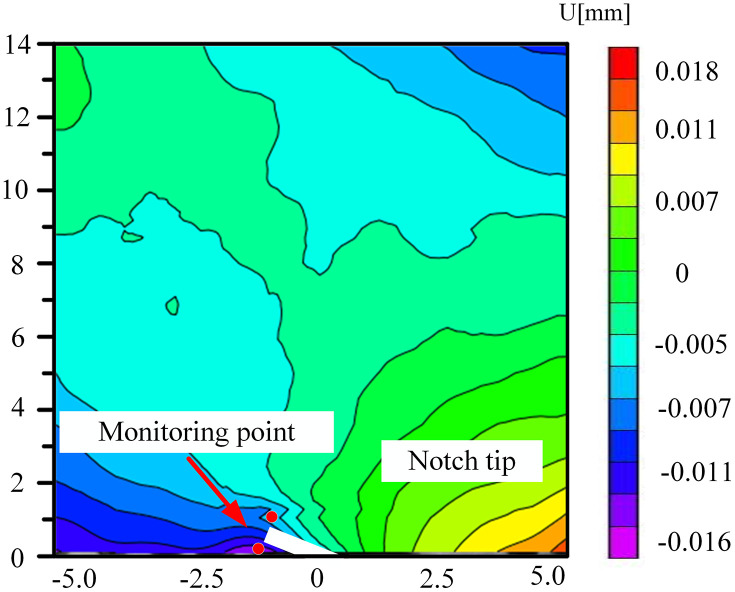
Horizontal displacement nephogram of 30°coal sample in microbial solution.

**Fig 33:**
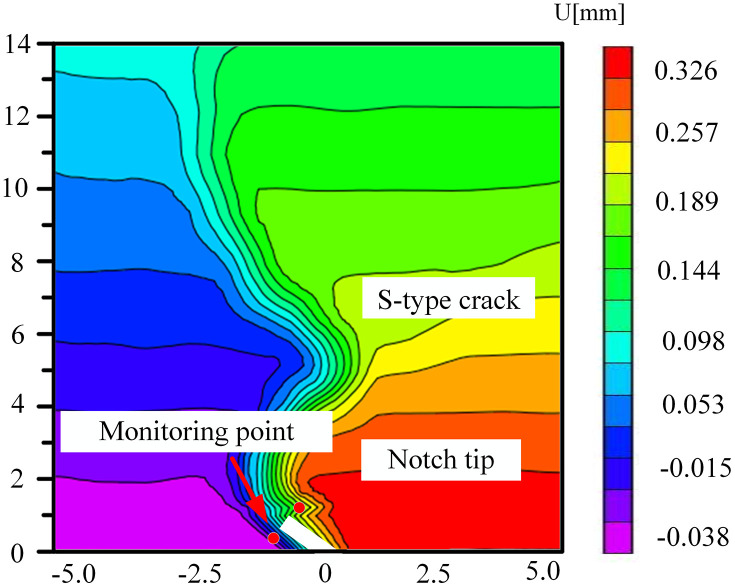
Horizontal displacement nephogram of 45°coal sample in microbial solution.

**Fig 34:**
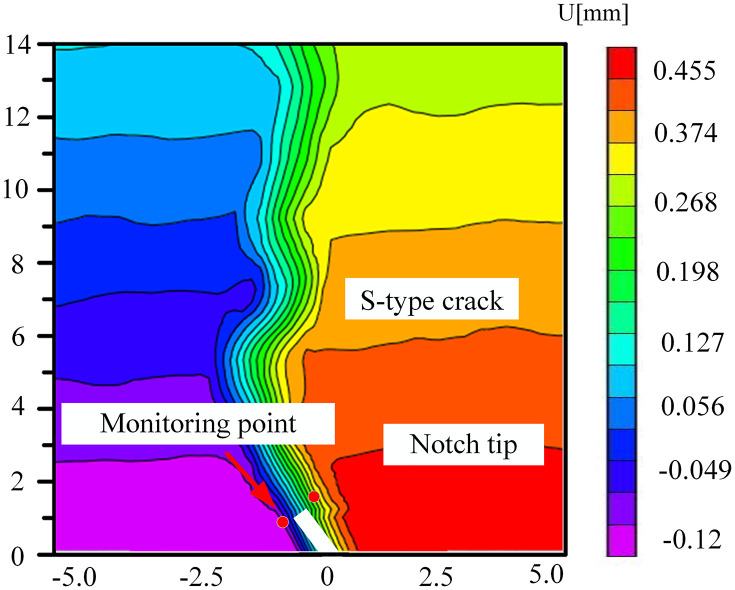
Horizontal displacement nephogram of 60°coal sample in microbial solution.

**Fig 35:**
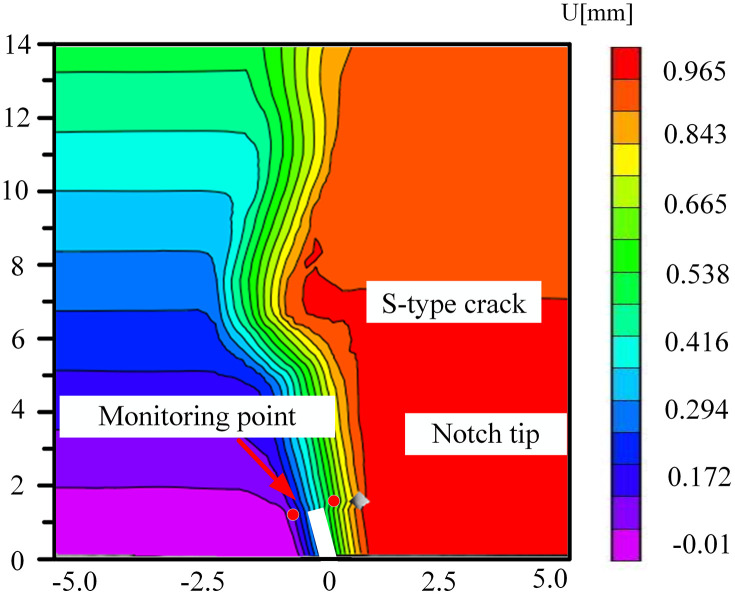
Horizontal displacement nephogram of 75°coal sample in microbial solution.

The accurate displacement-change trend of the horizontal displacement field of the 30° specimen under different solutions at the peak load could not be observed. This might be attributed to the fact that the crack dip angle was nearly perpendicular to the loading direction. During loading, the stress might have been preferentially released along the primary crack, causing the main crack not to initiate from the prefabricated tip. Consequently, the DIC displacement field exhibited a multi-region scattered transition rather than a continuous gradient change. The crack-propagation stage during the brittle fracture of the coal sample was too rapid. The algorithm might not have been able to accurately resolve the displacement field, manifested as abnormal or missing data.

## 6. Conclusion

1. During the soaking process of anthracite, the functional groups and side chains fall off, the benzene ring opens, the macromolecular structure is broken, the secondary micro-cracks and holes are formed, and the organic acids such as acetic acid are produced to dissolve the calcite and other minerals filling the pores, and the natural cracks are reopened. Microbial action significantly changed the pore distribution pattern and reduced the strength of coal. The chemical reaction between methanogens and coal samples uses microorganisms to degrade part of coal into biogas dominated by methane, which not only increases the amount of coalbed methane resources, but also achieves biological permeability enhancement.
2. With the increase of crack angle, the average value of peak load, peak displacement and fracture toughness of anthracite decreased from 0.223 to 0.191 Mpa.m^1/2^, and the fracture toughness of coal samples decreased gradually. The core mechanism is that the relative relationship between the crack dip angle and the loading direction changes the stress distribution pattern at the crack tip. It shows that when the loading direction is close to the crack angle, the fracture toughness is the smallest, and when the loading direction is close to the crack angle, the fracture toughness is the largest. The heterogeneity of coal will also affect the failure mode.
3. The crack tip of SCB specimen is mainly tensile deformation, supplemented by shear failure. The intervention of microbial solution further weakens the tensile strength of coal. The tensile failure range under neutral solution immersion is 57.97 ~ 86.7% P_max_, and the initial time range of microbial tensile deformation is 36.36 ~ 60.52% P_max_. The tensile deformation of the crack tip under the microbial solution is prior to the tensile deformation under the neutral solution, and the length of the crack propagation process zone (FPZ) generally increases by about 37.5%.

## Supporting information

S1 File S1 raw images. This is the Sl File legend.
(PDF)
