# Supplementary figures and images for: Study on fracture of coal samples with different fracture angles under microbial environment
Source: PLoS One. 2025 Nov 6;20(11):e0333227. doi: 10.1371/journal.pone.0333227 (PMC12591433; doi:10.1371/journal.pone.0333227)

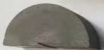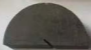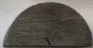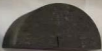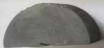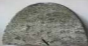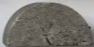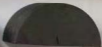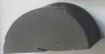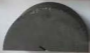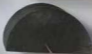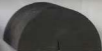

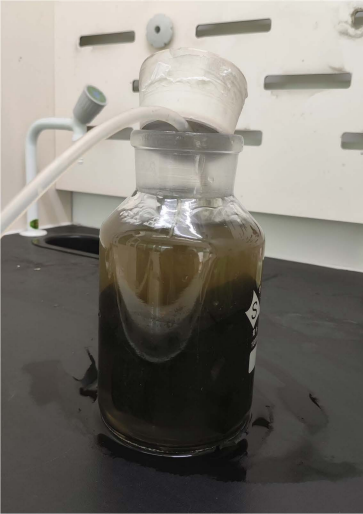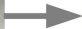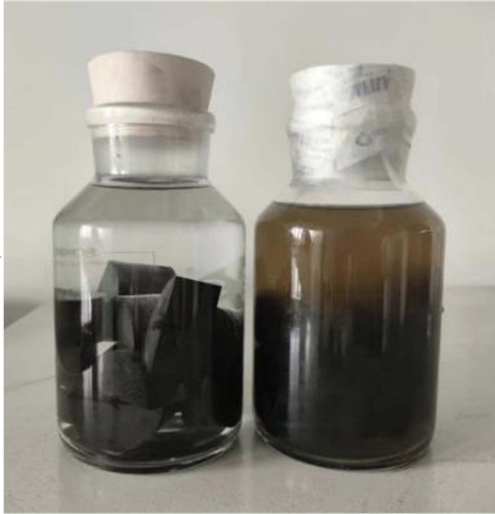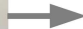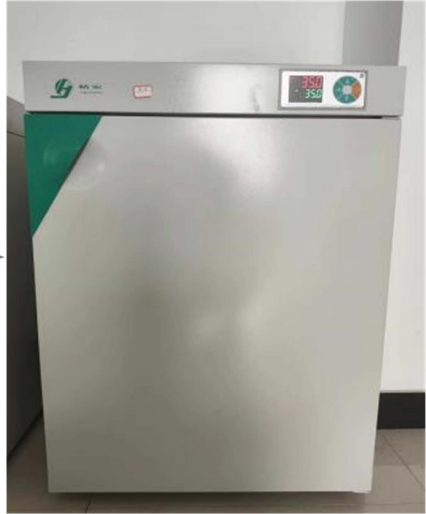

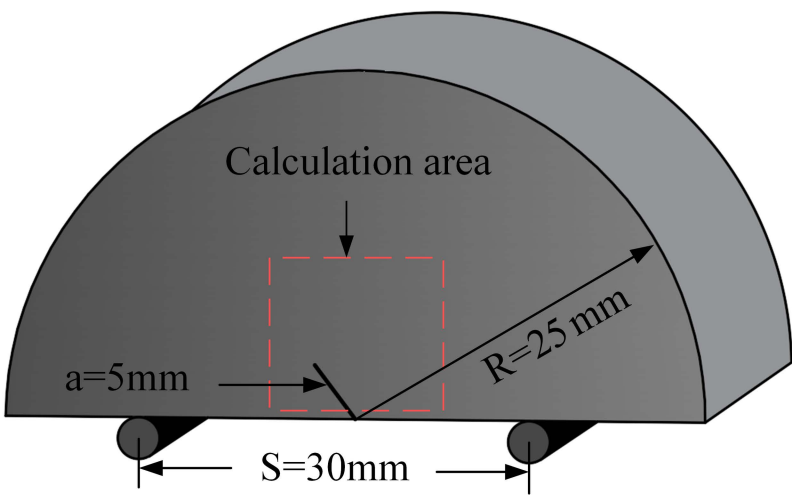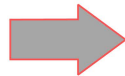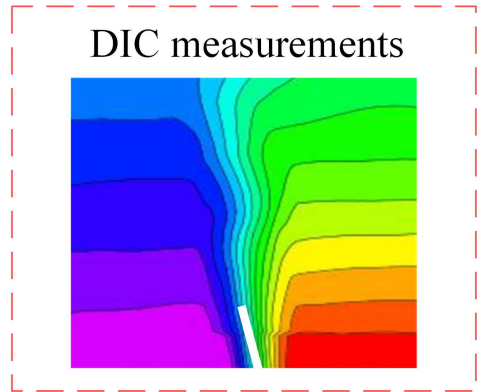

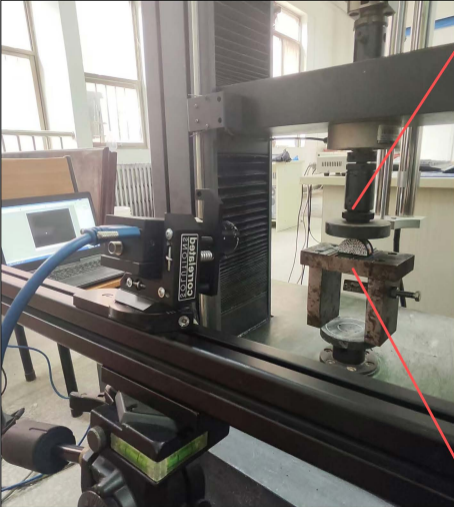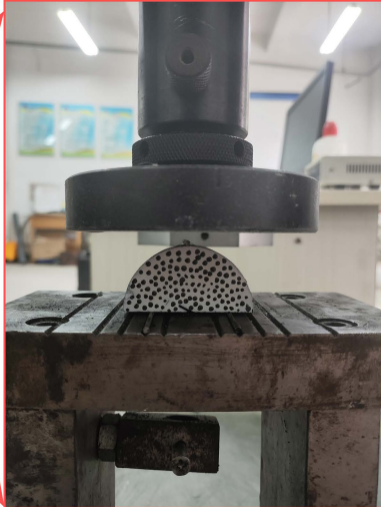

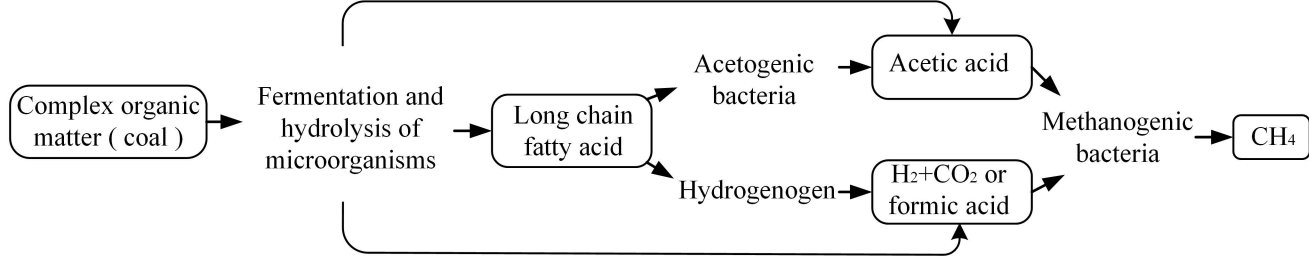

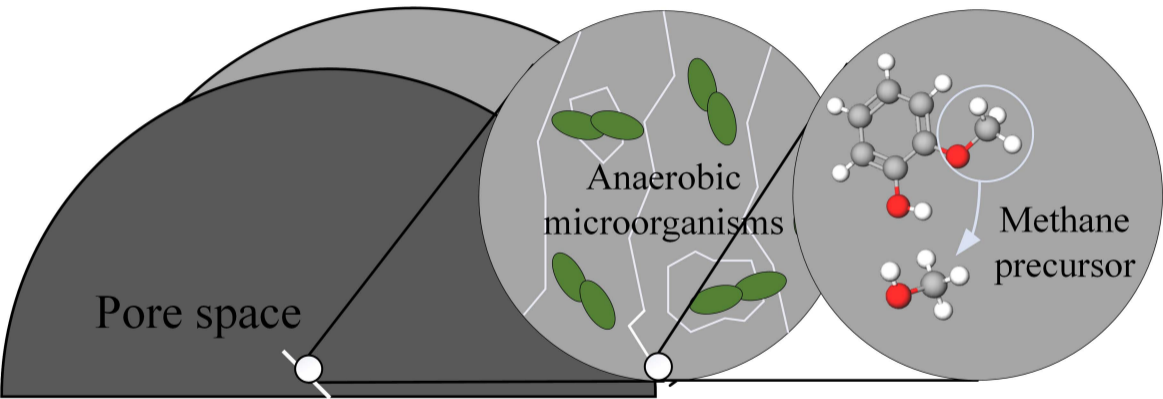

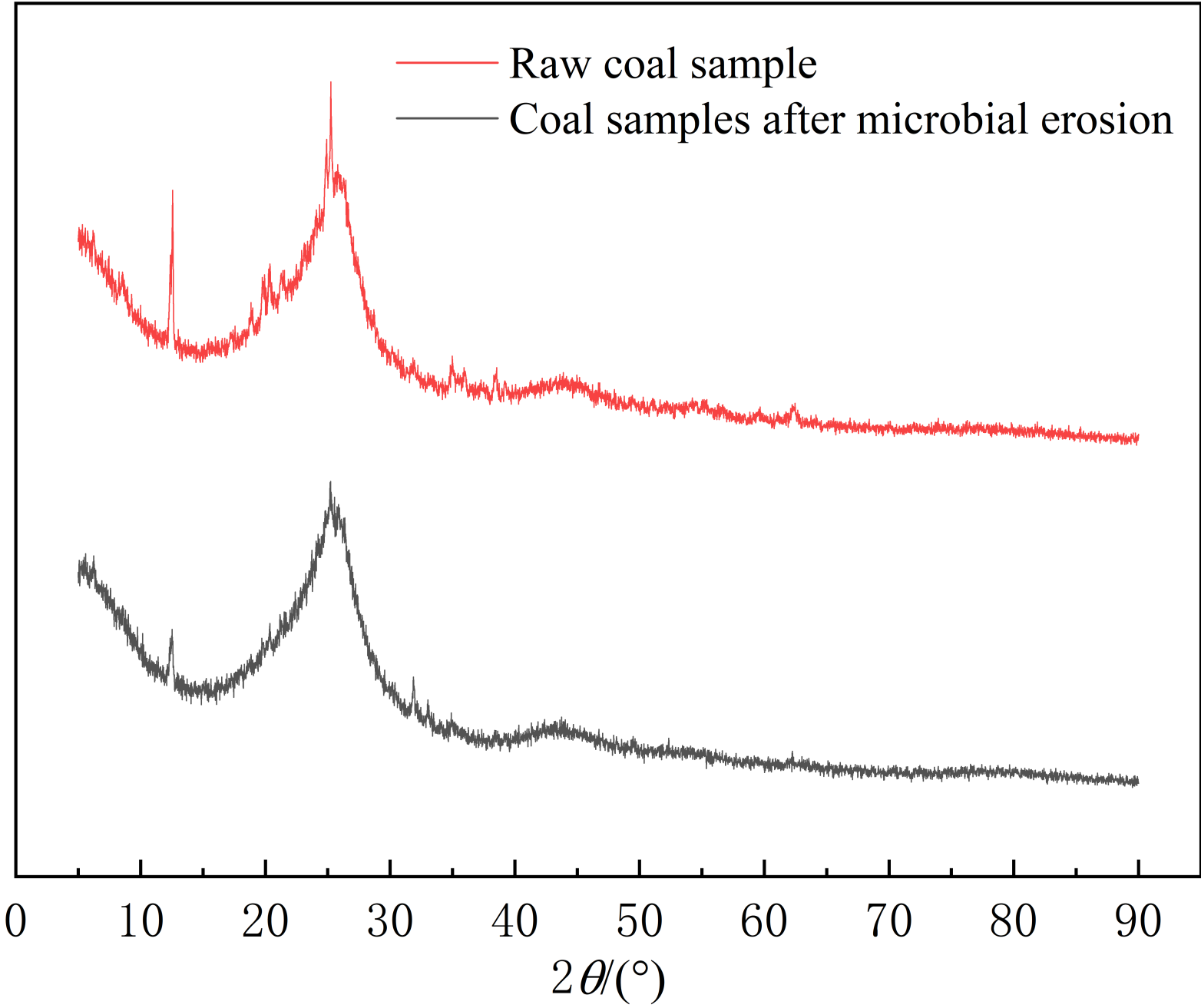

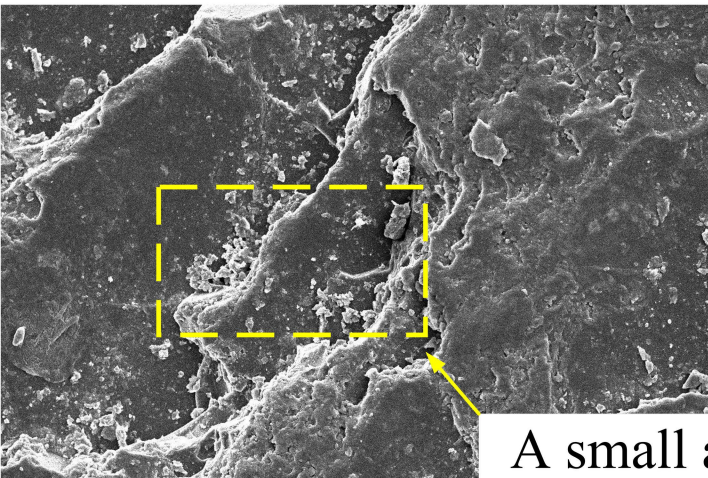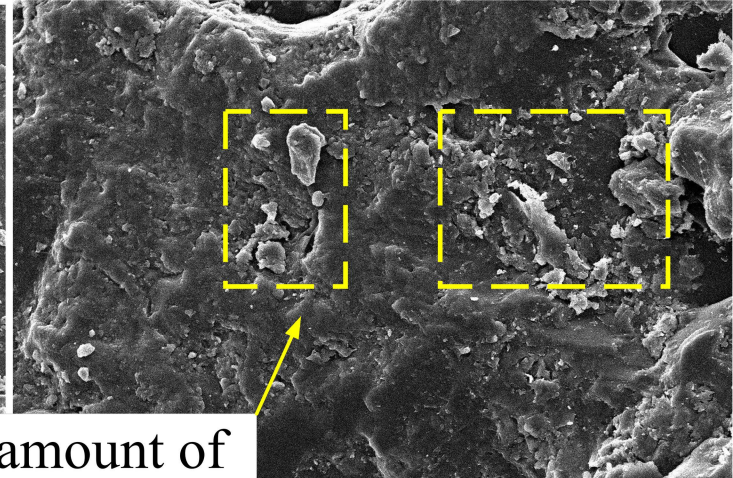

A small amount of  
holes, particles

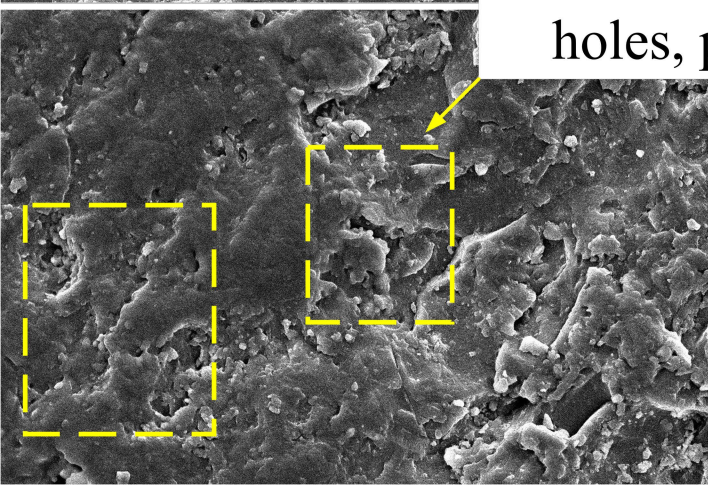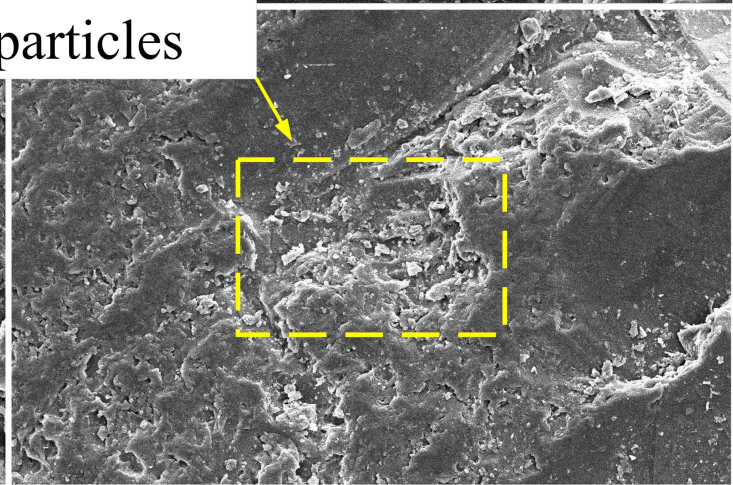

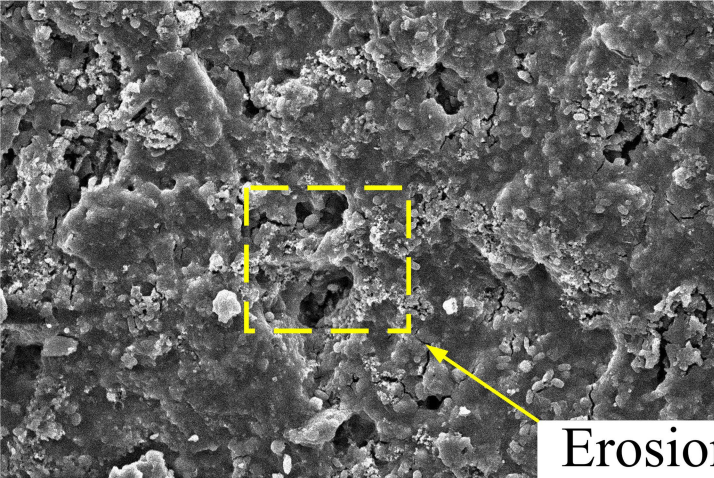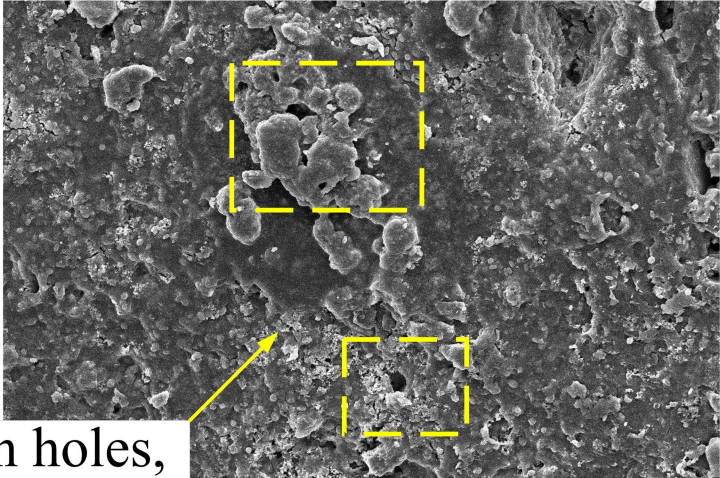

Erosion holes,  
cracks

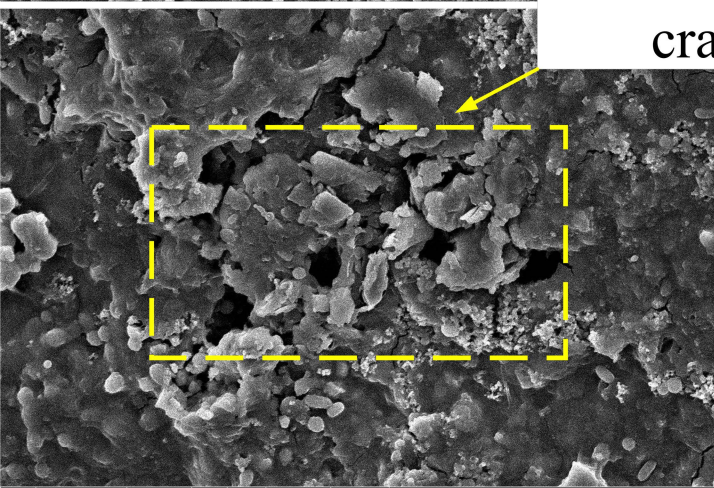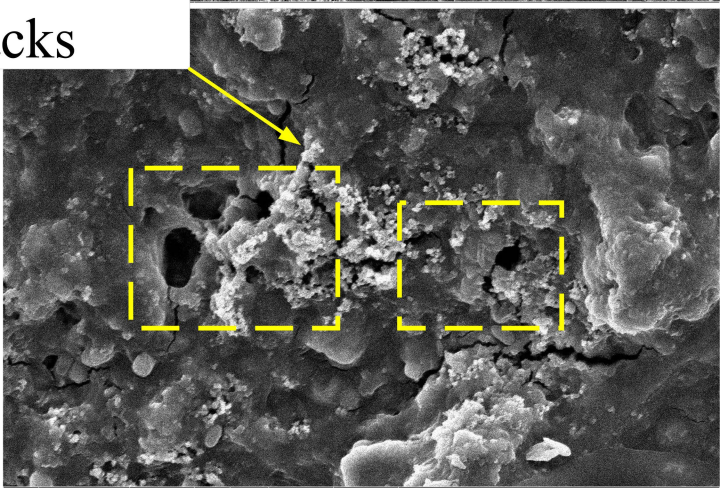

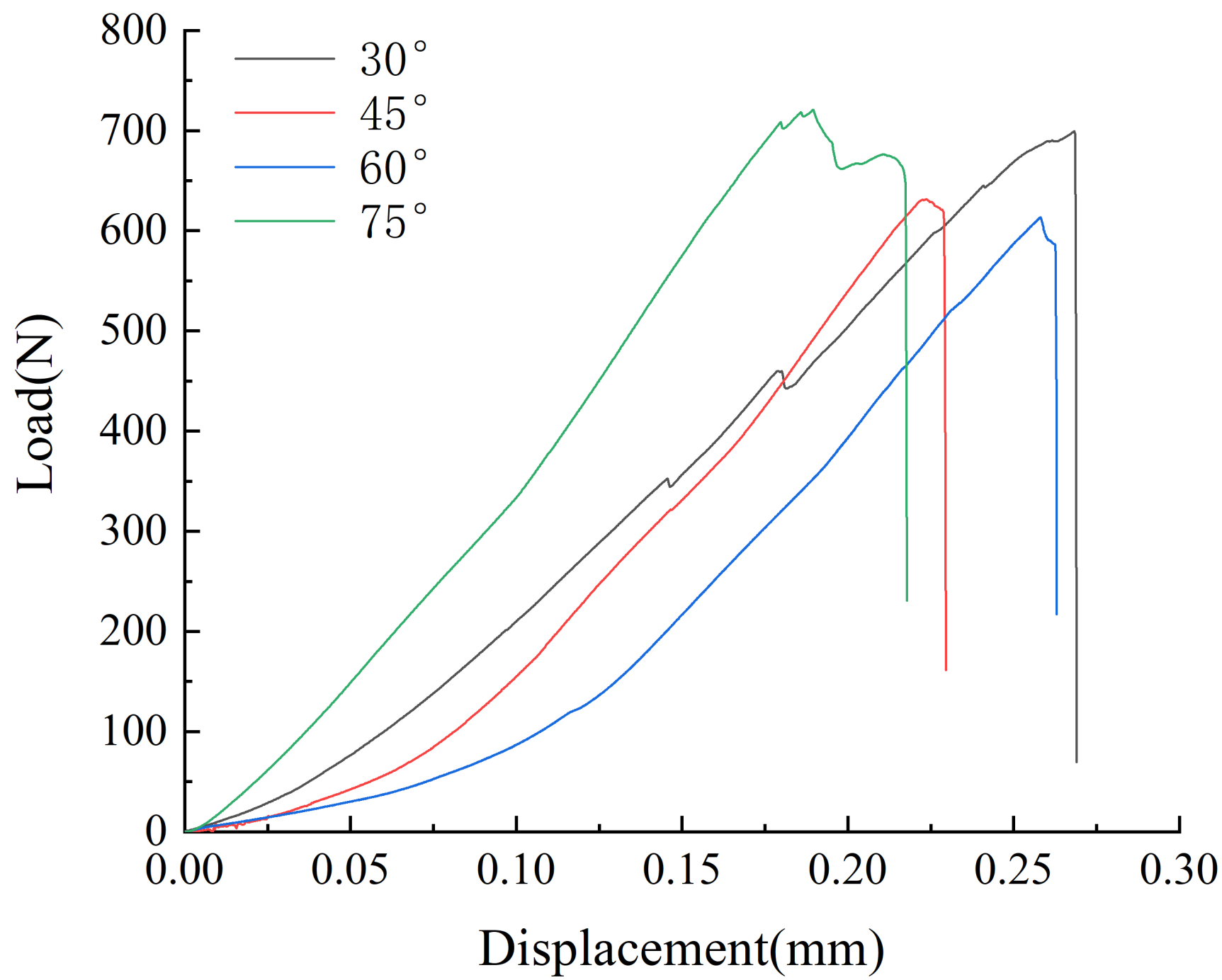

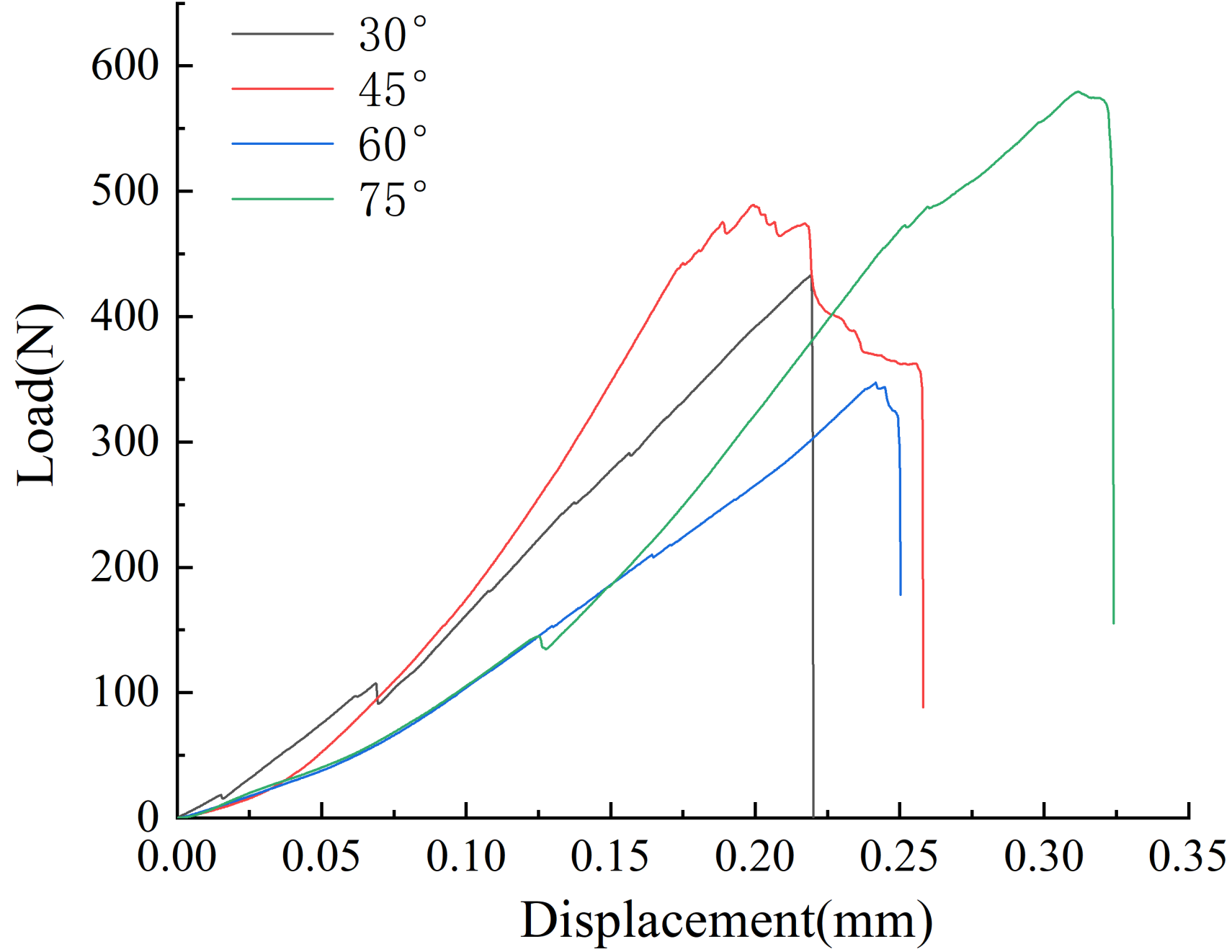

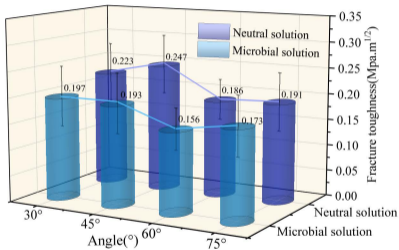

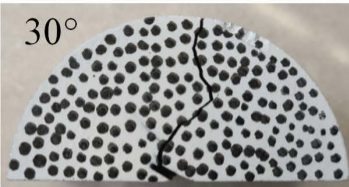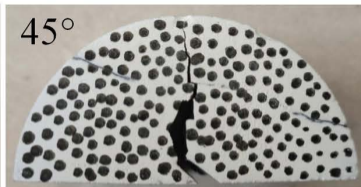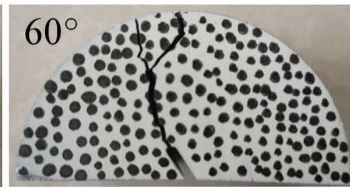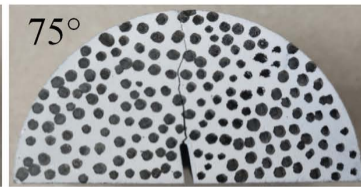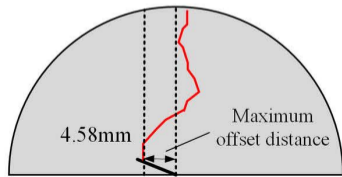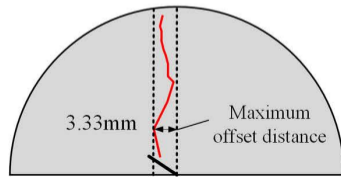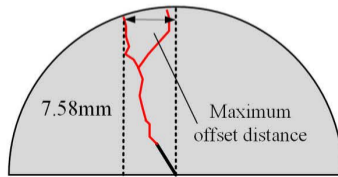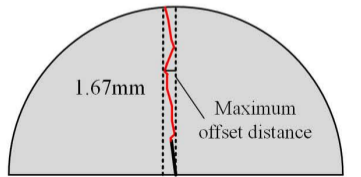

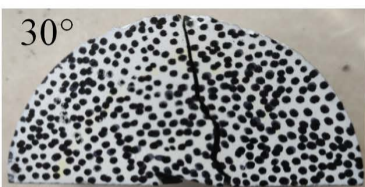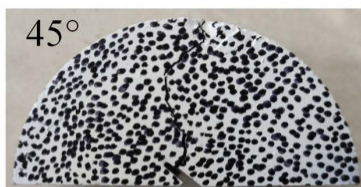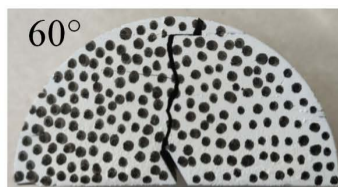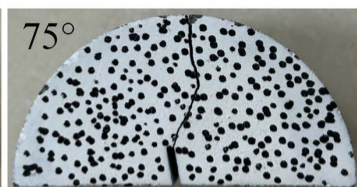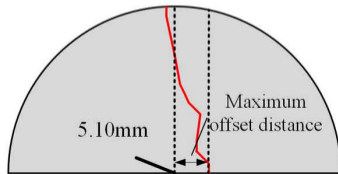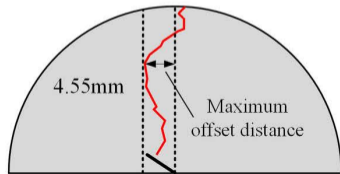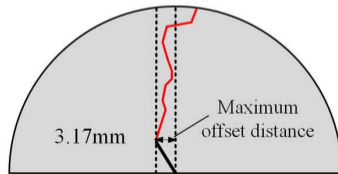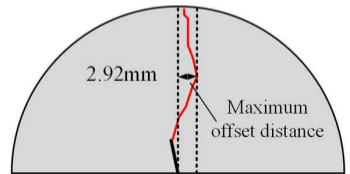

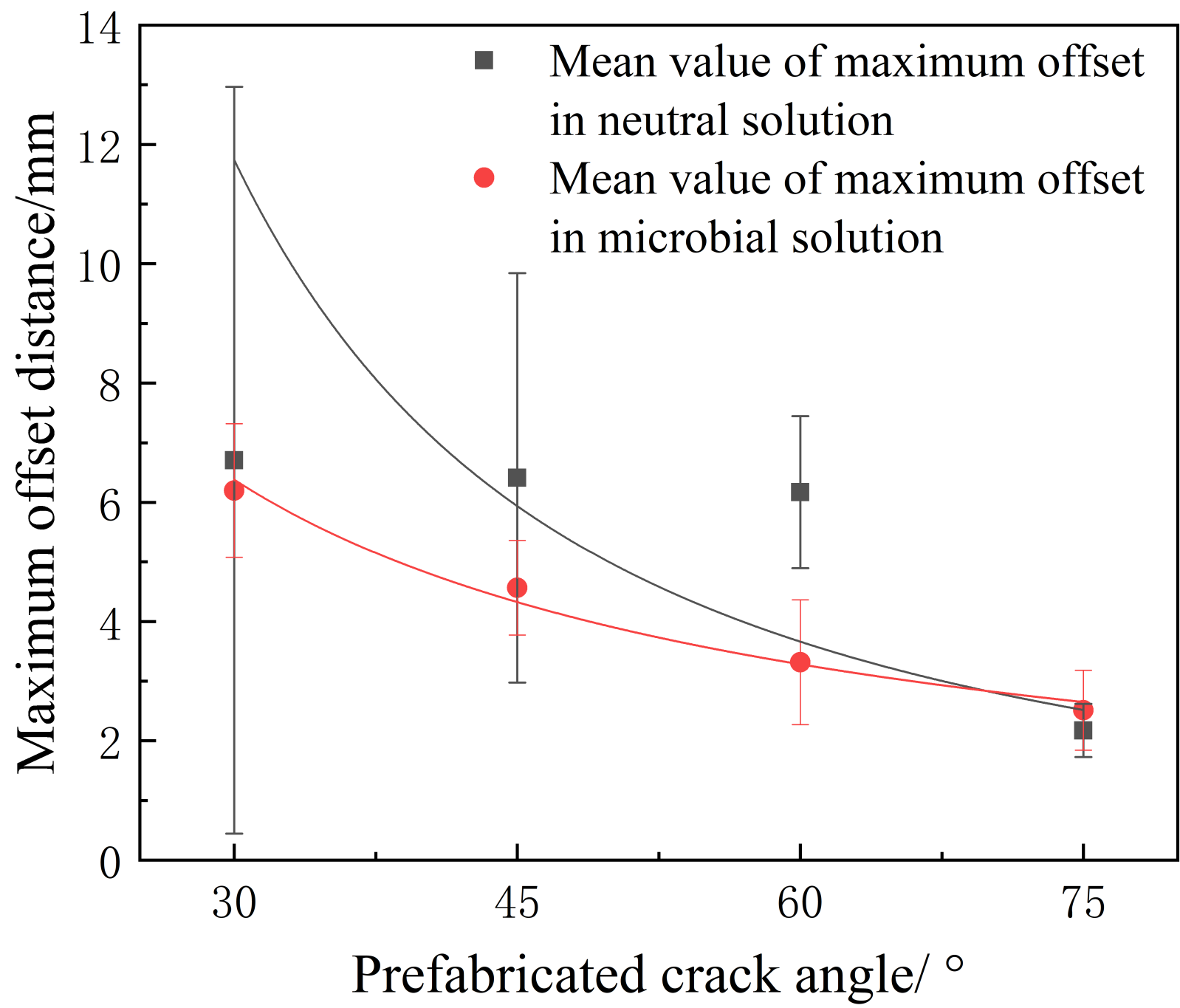

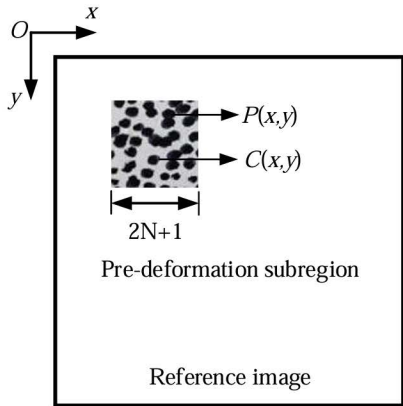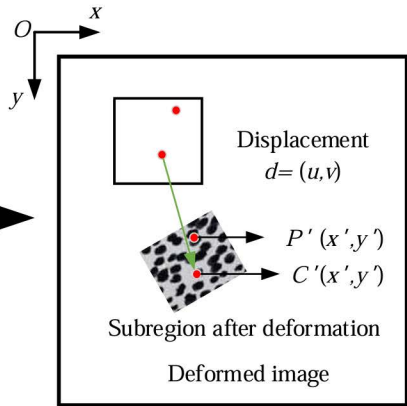

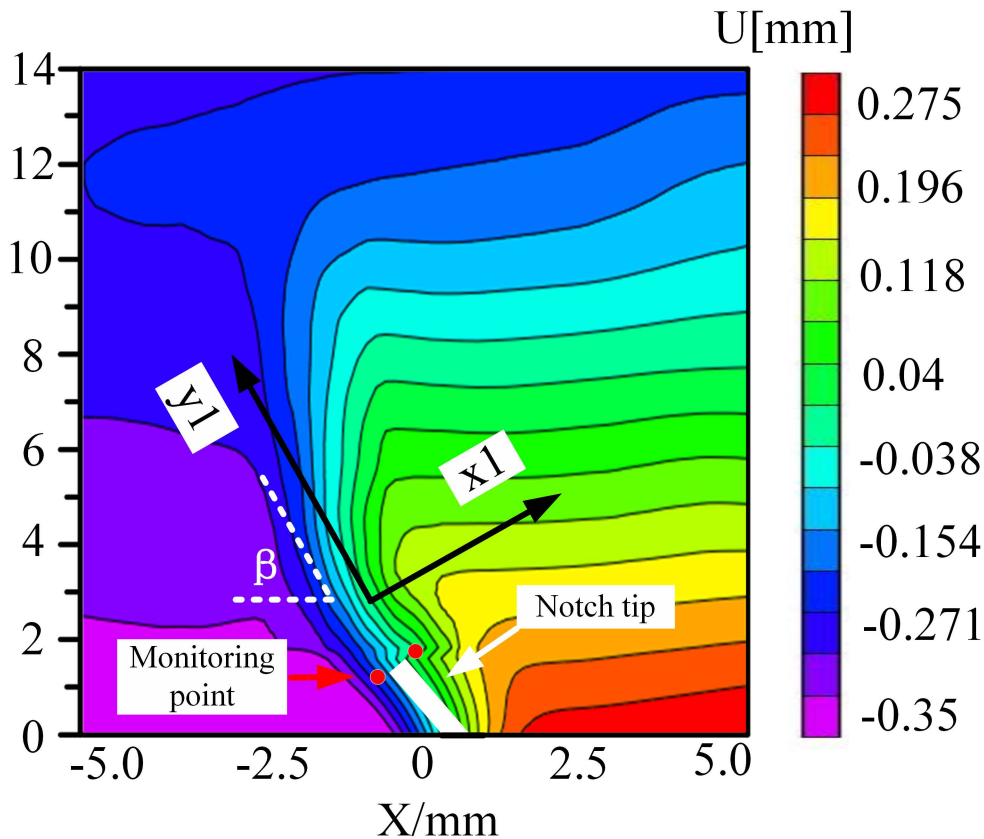

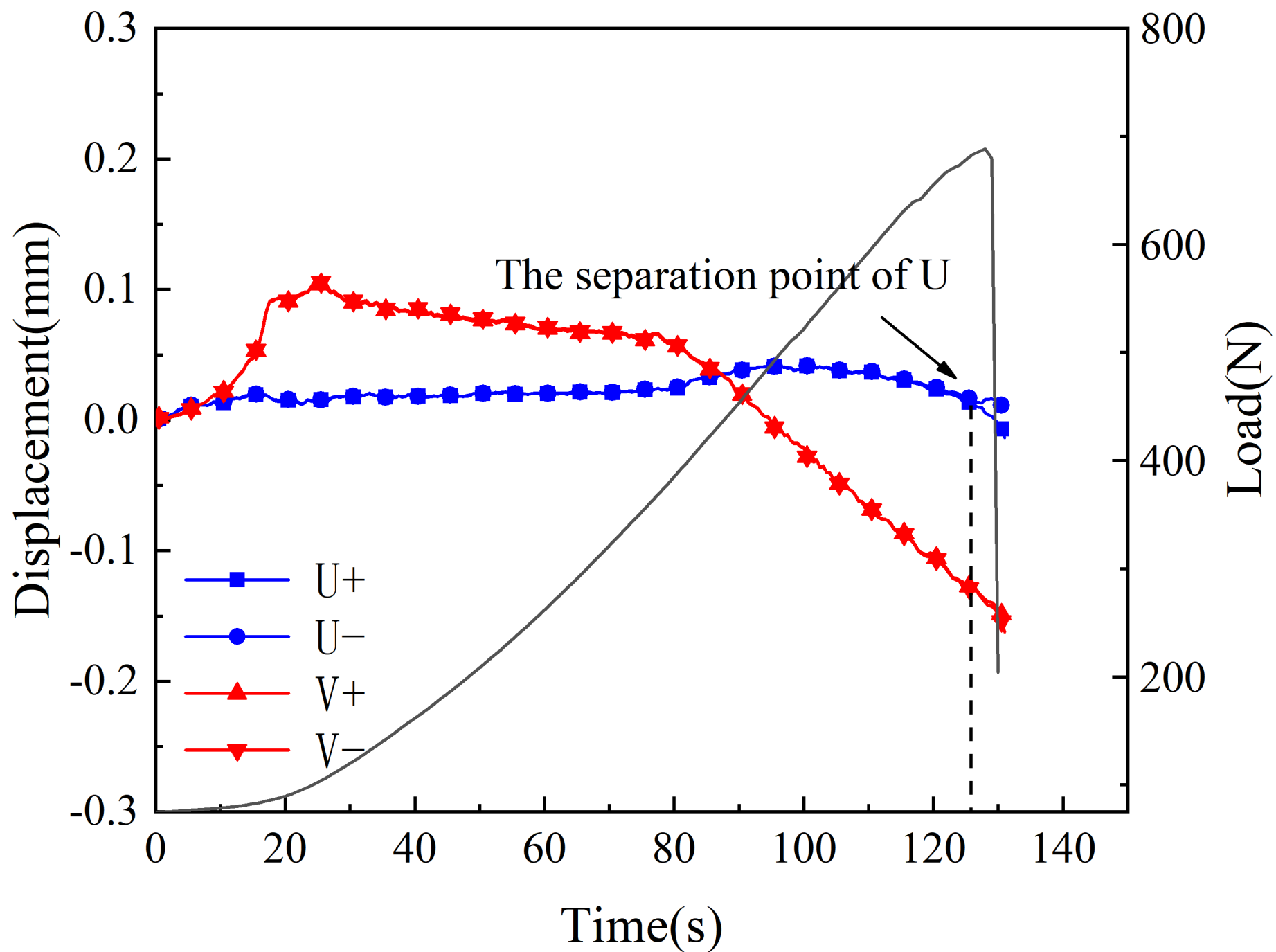

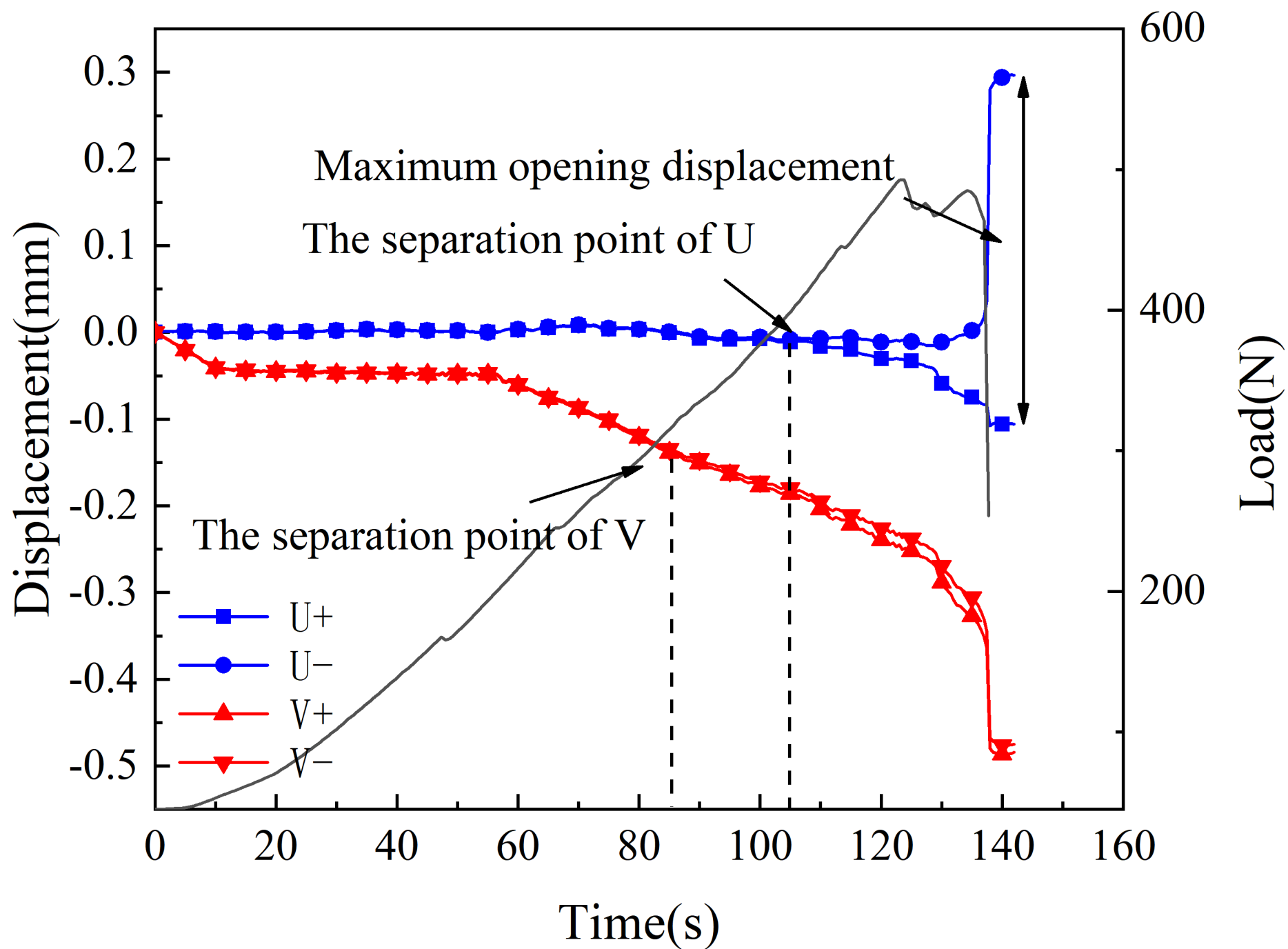

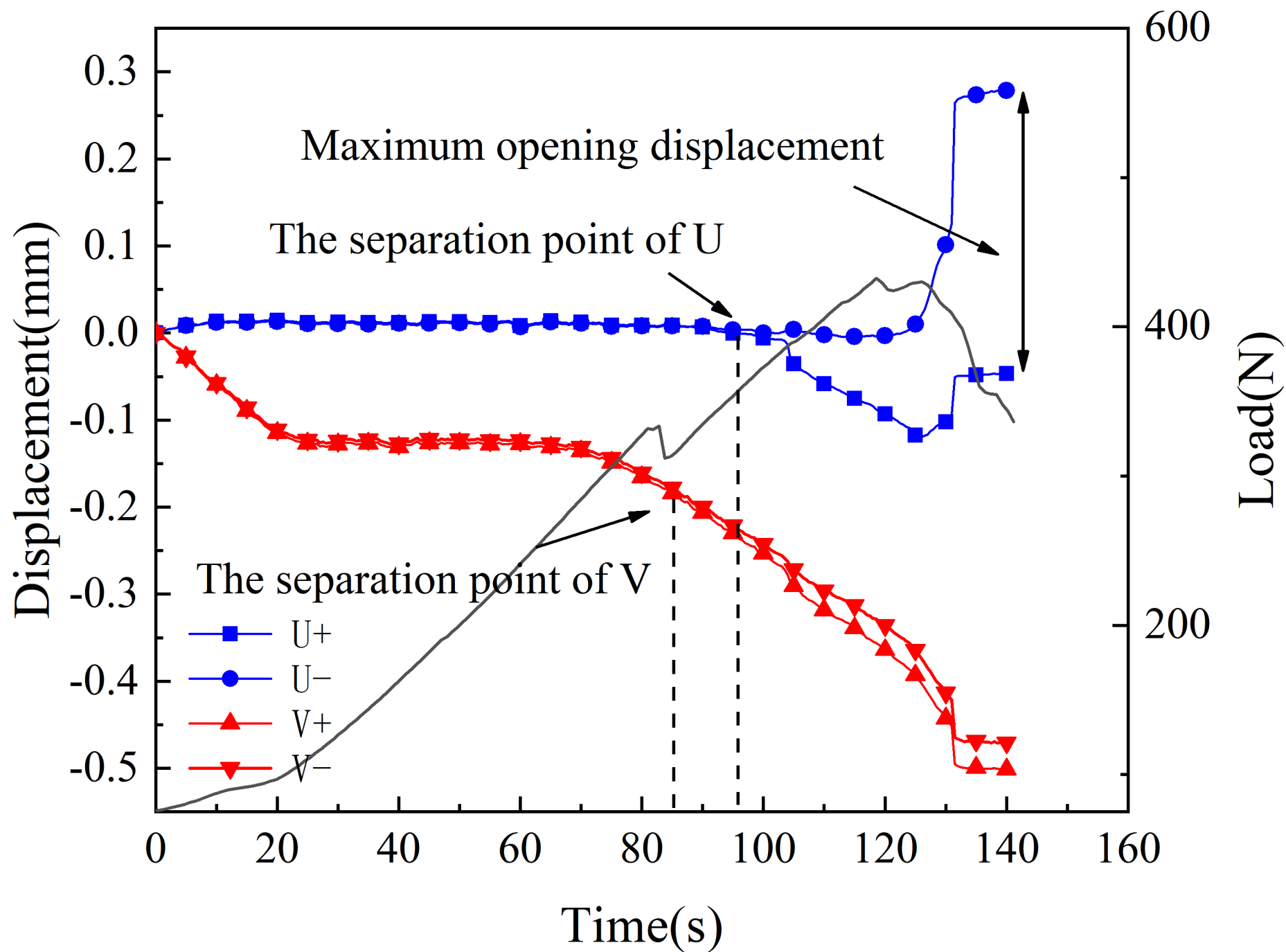

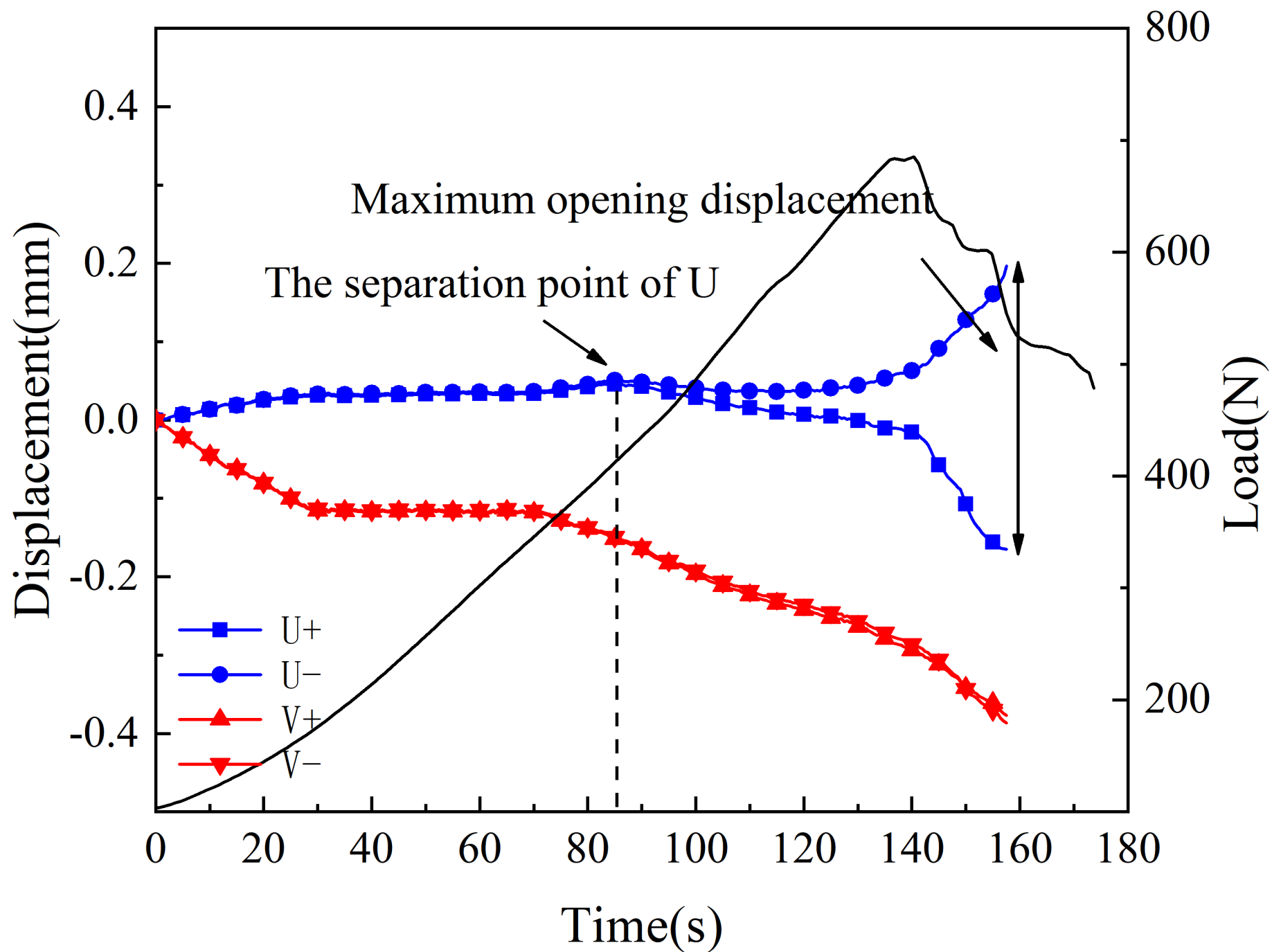

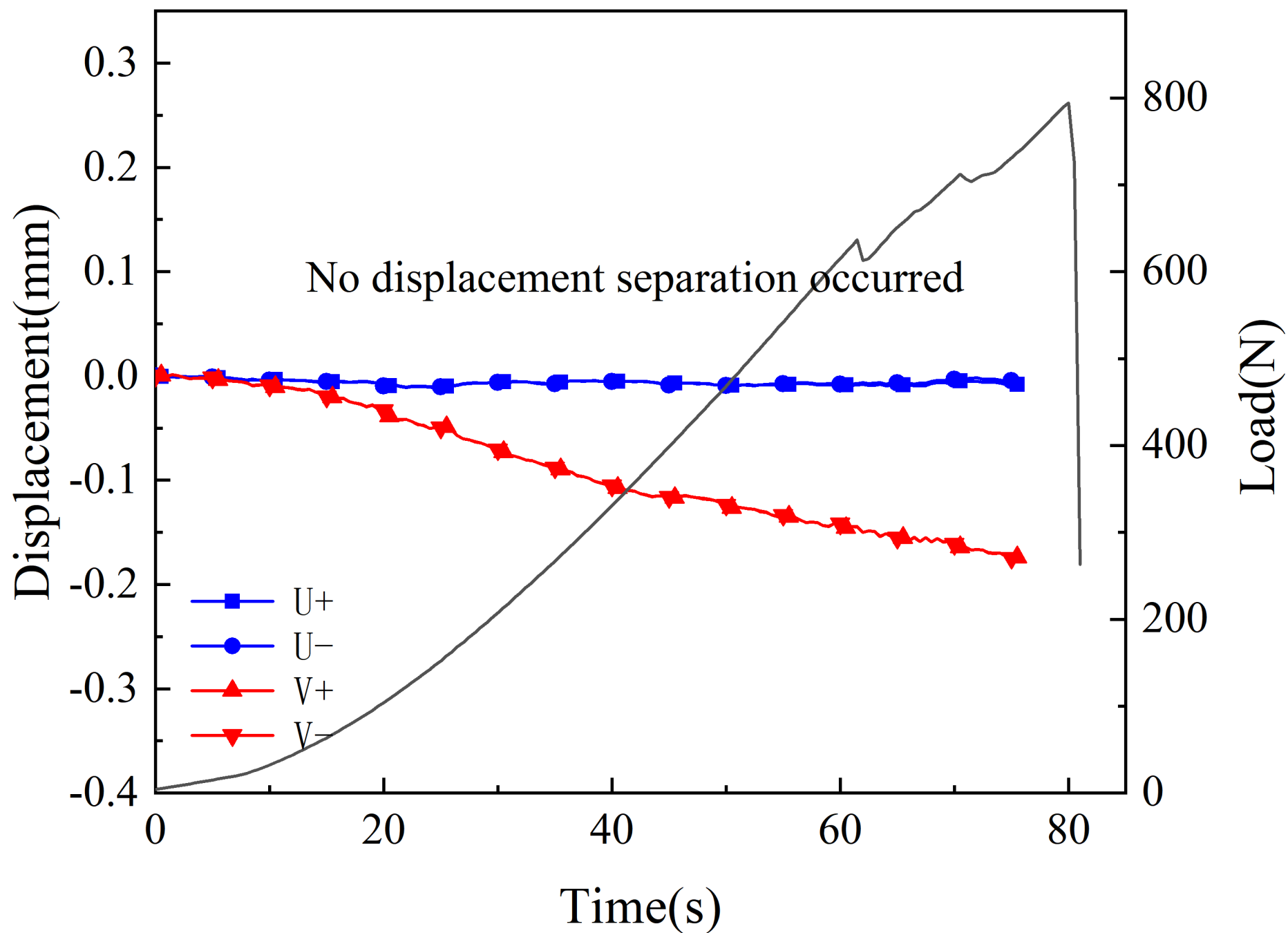

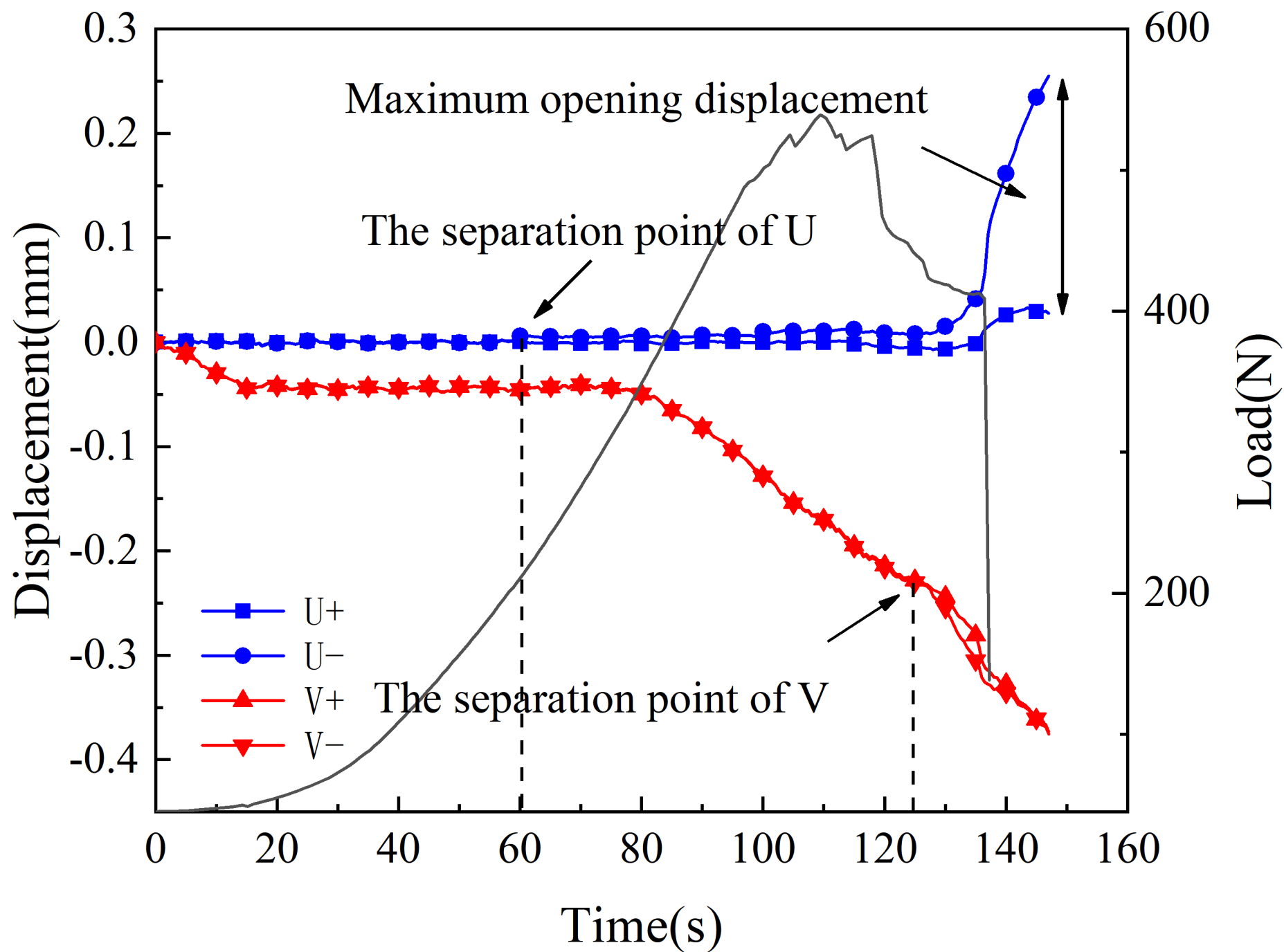

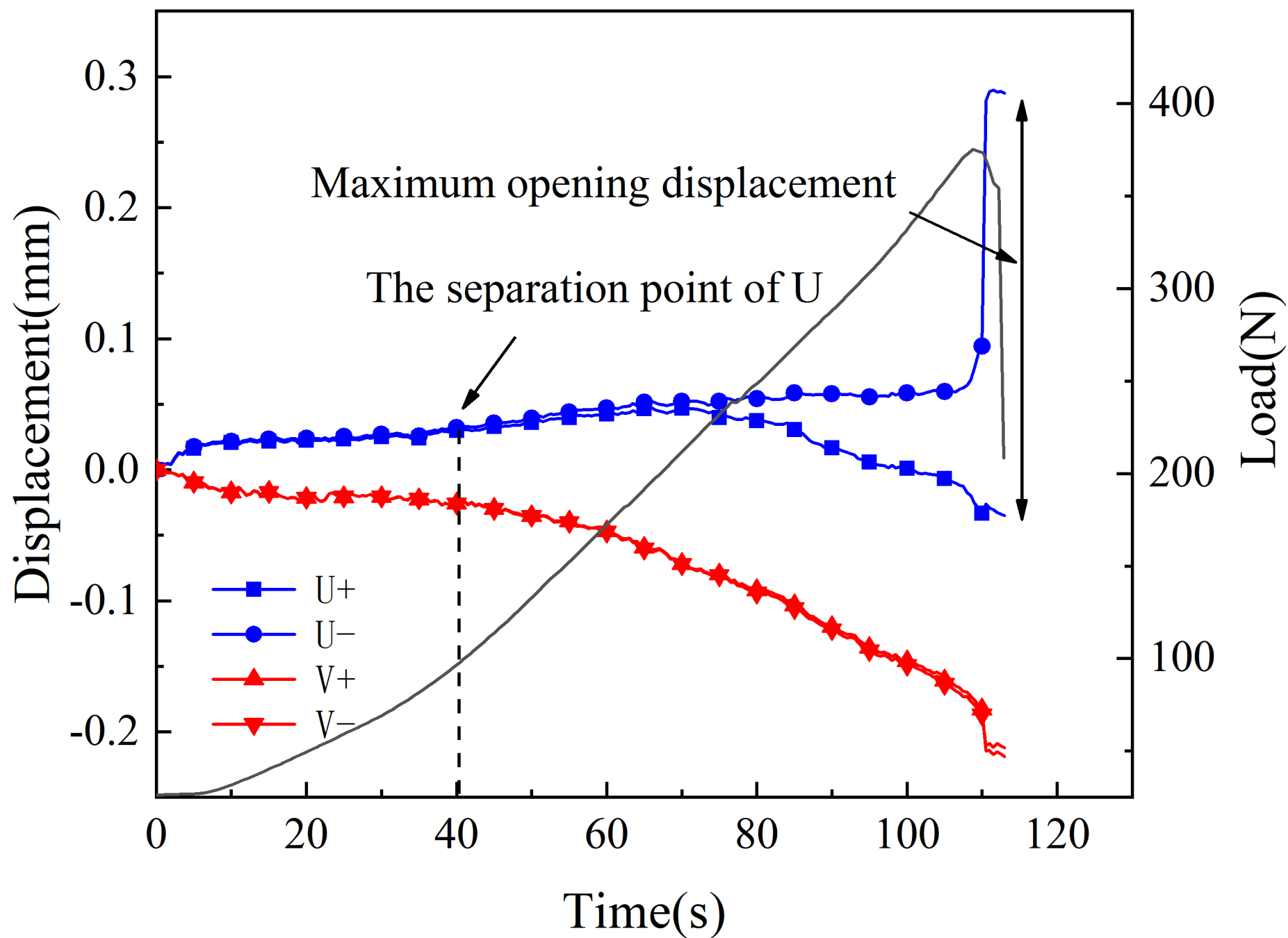

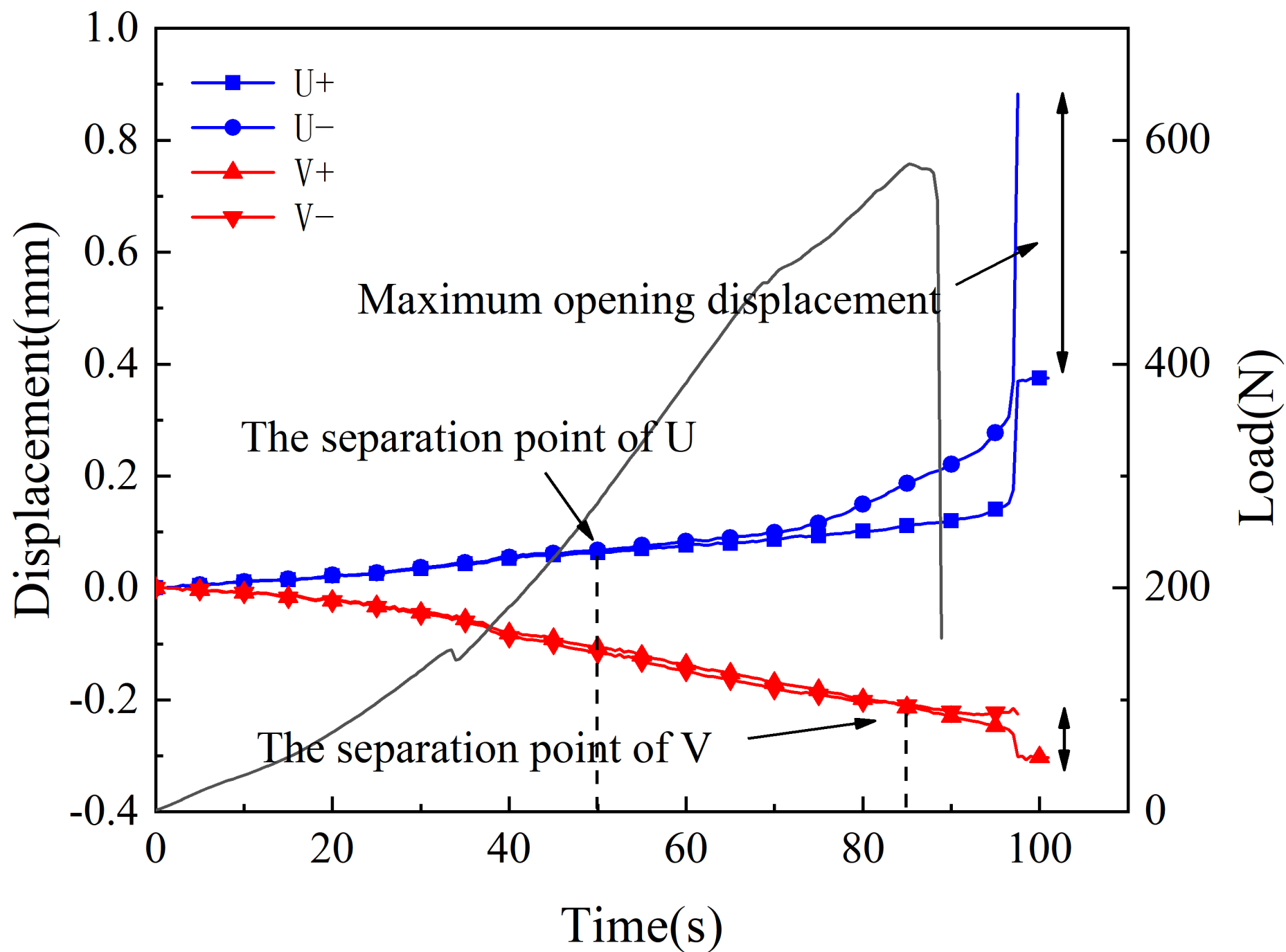

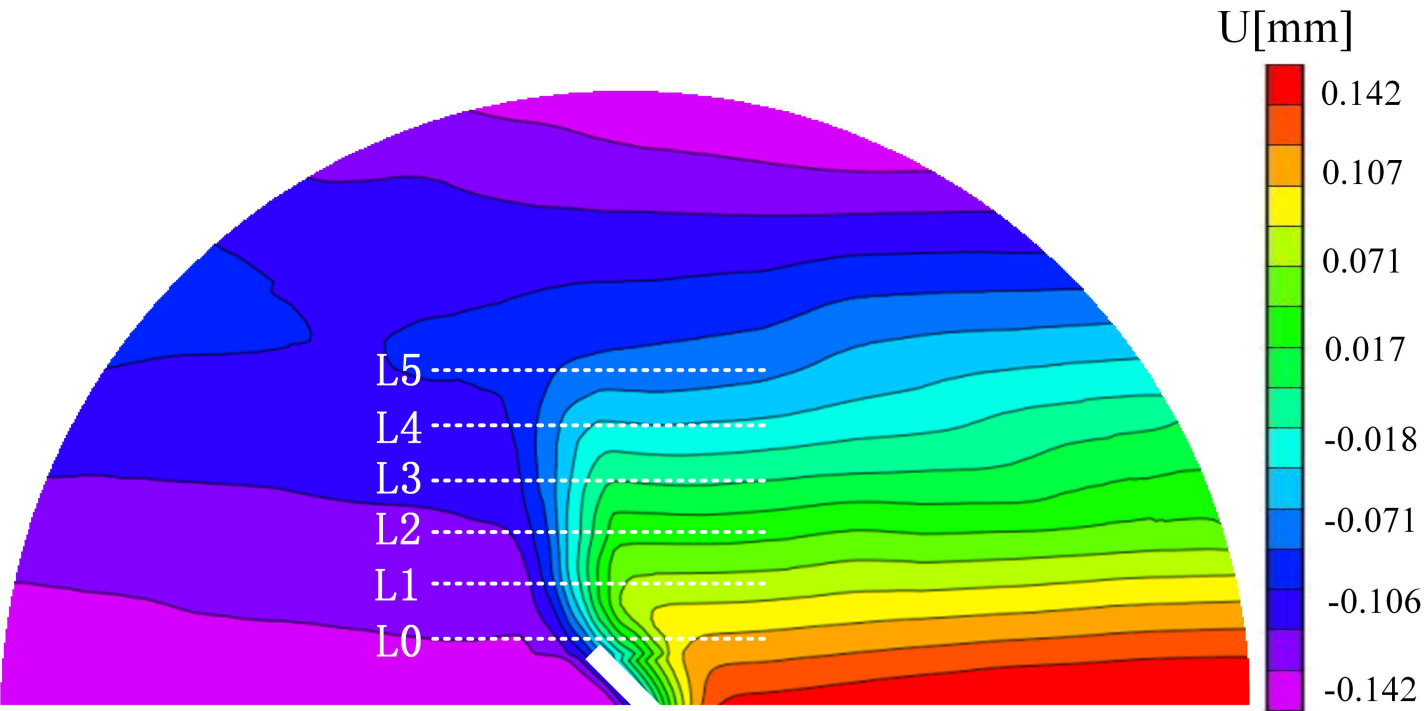

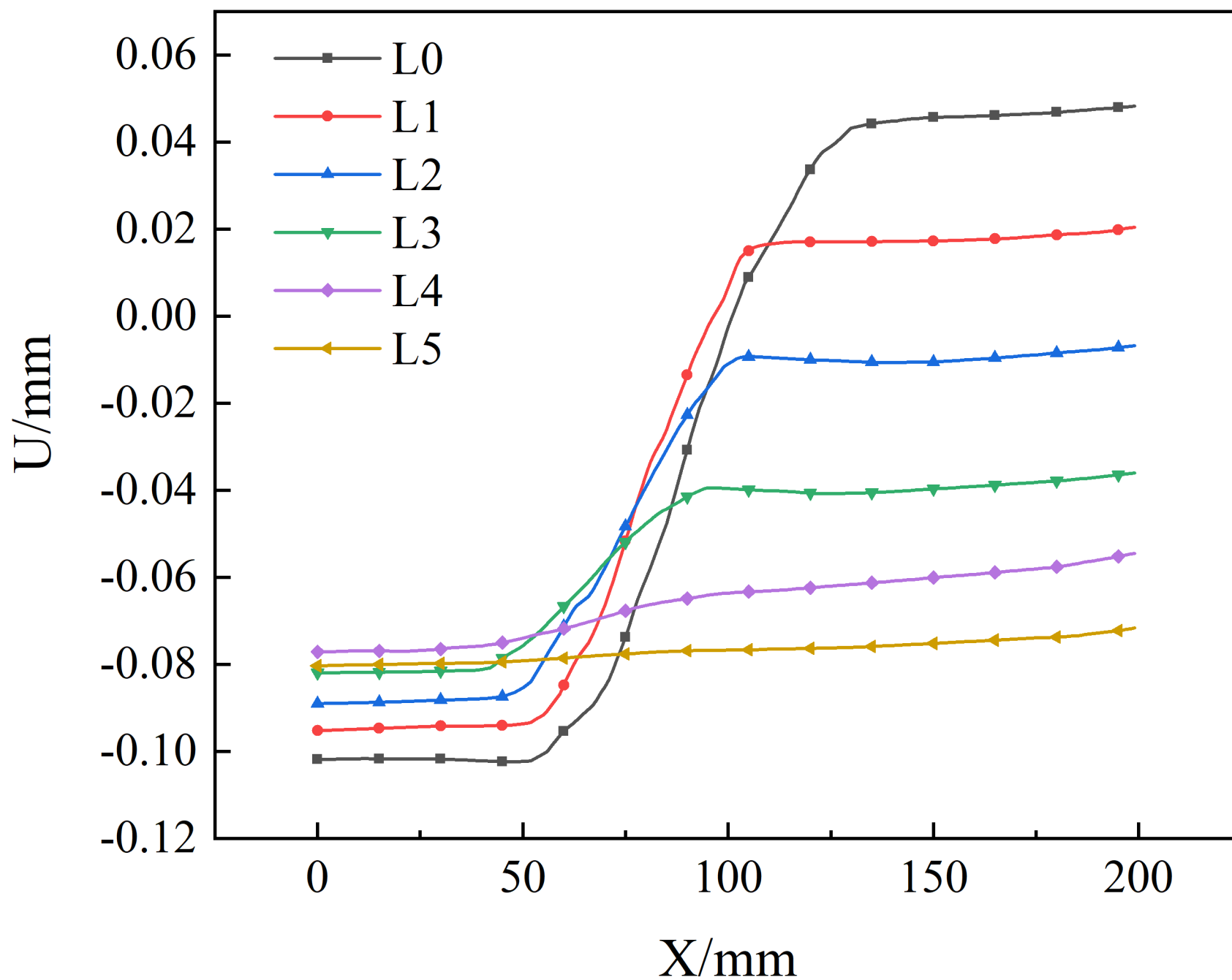

U[mm]

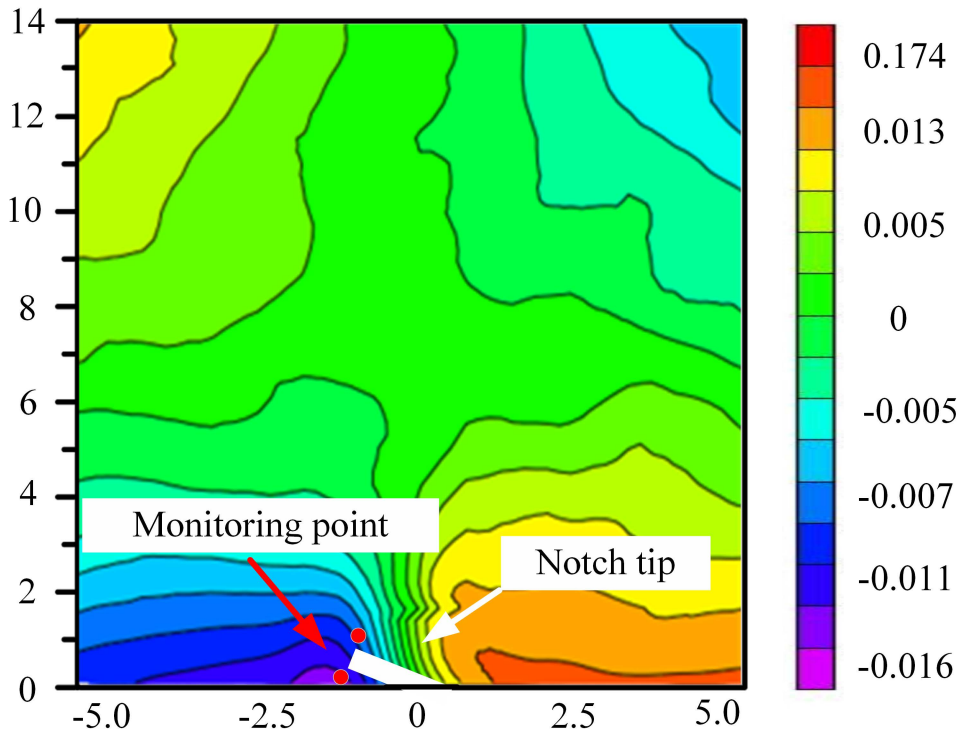

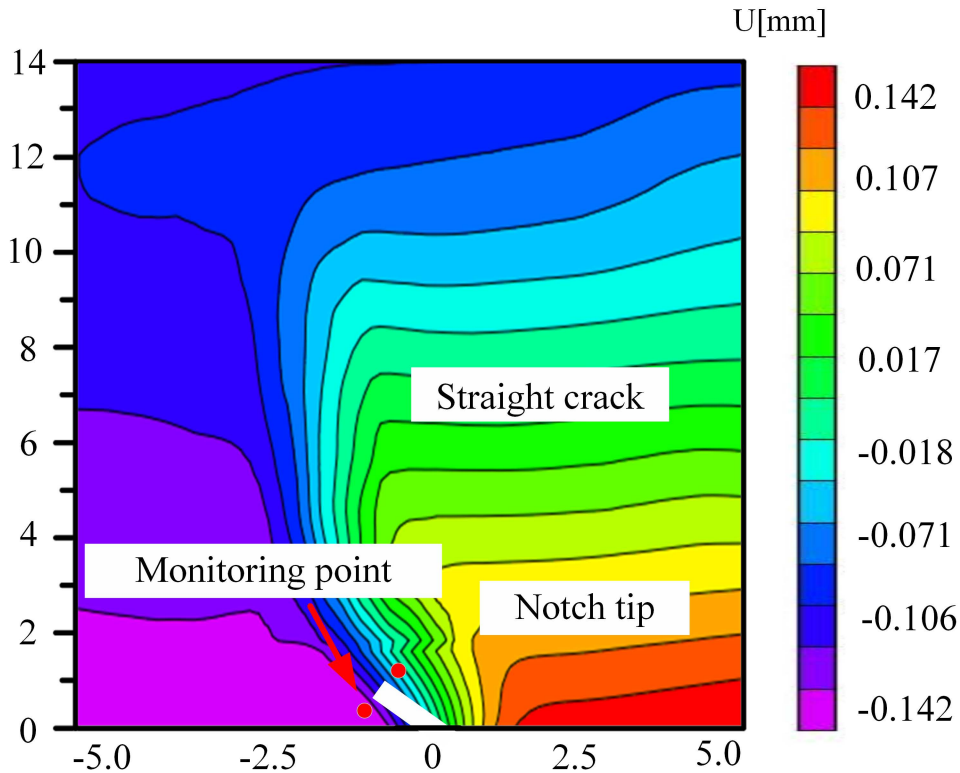

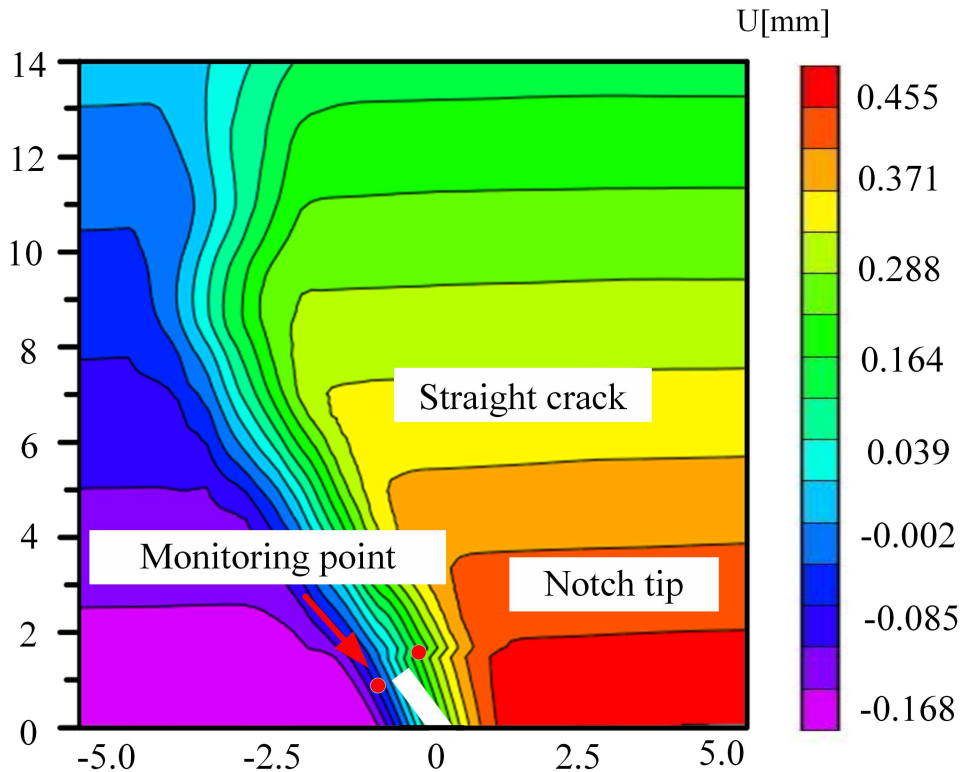

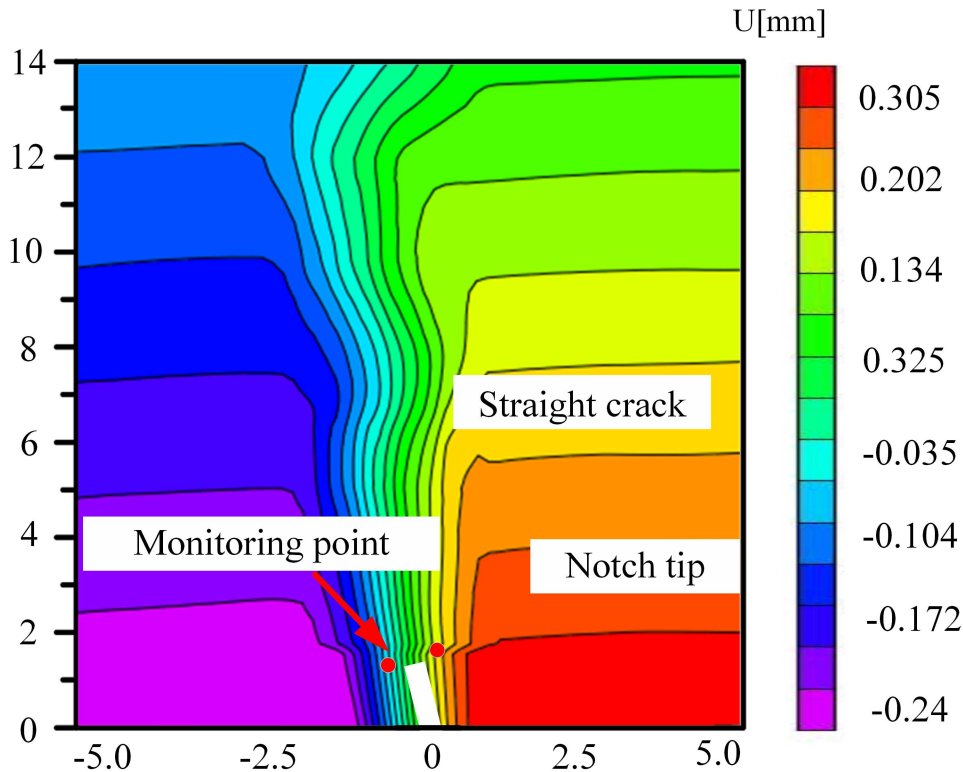

$U[\text{mm}]$

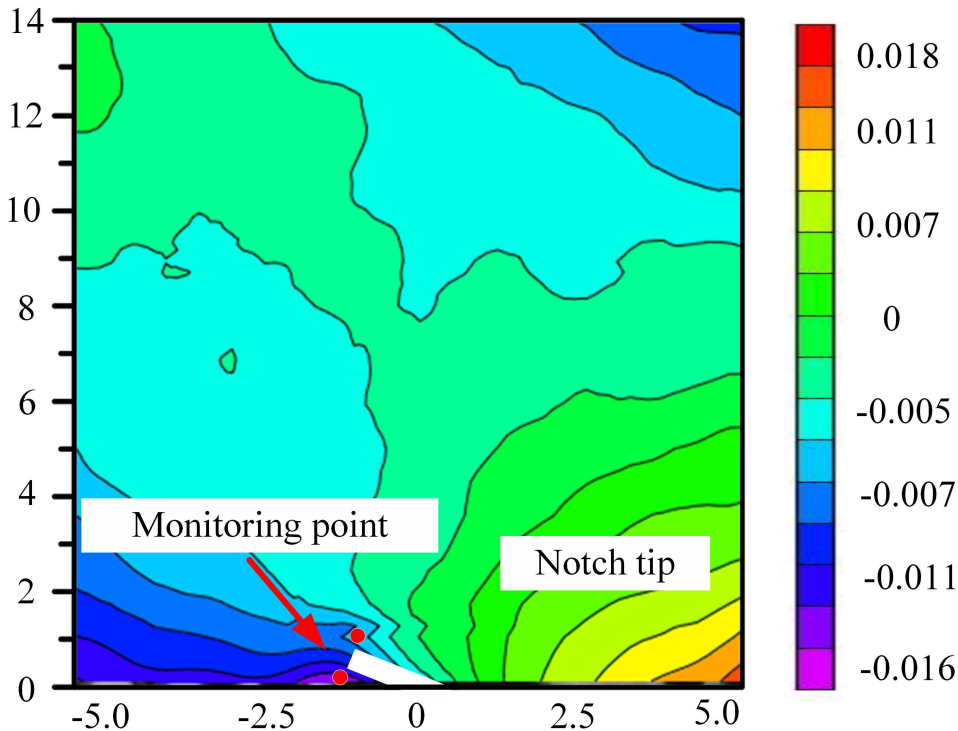

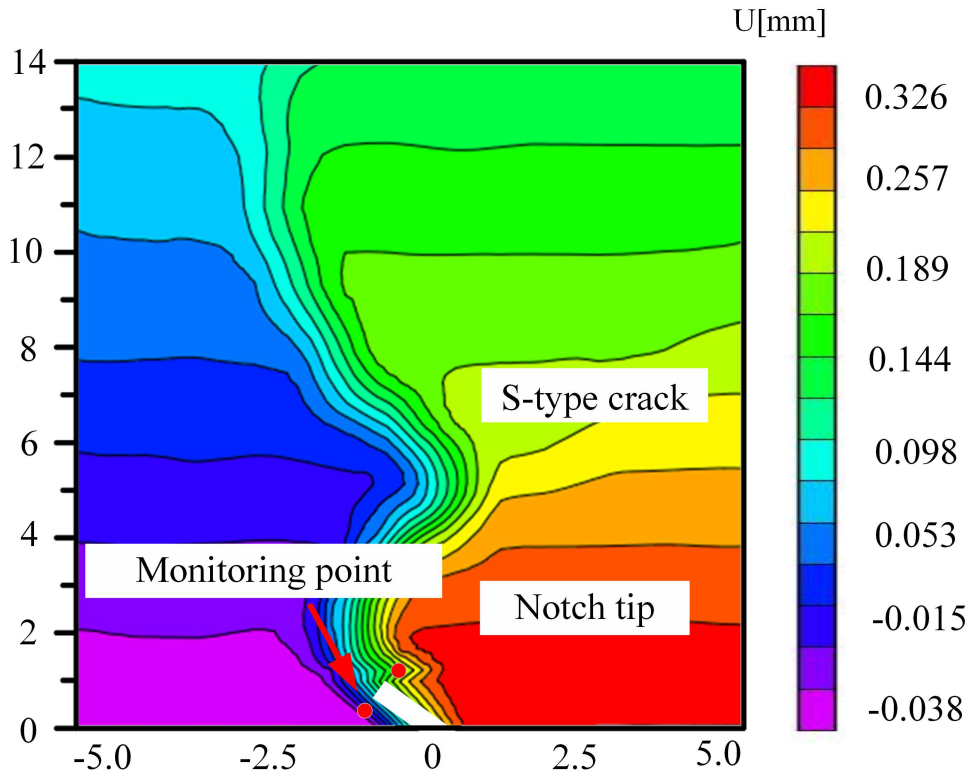

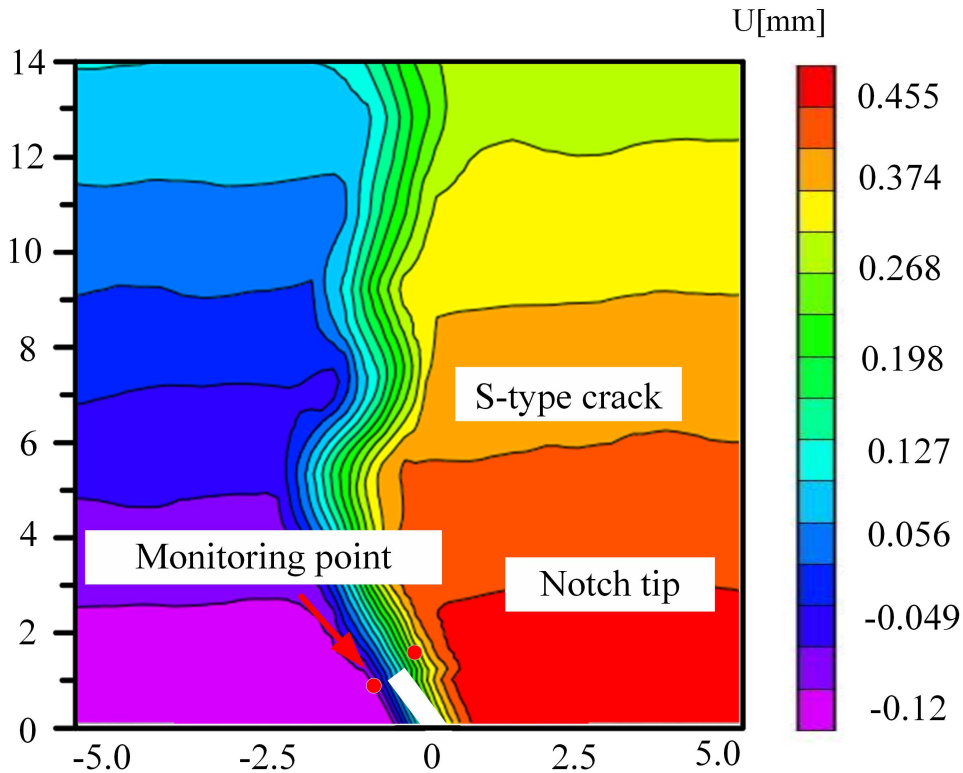

U[mm]

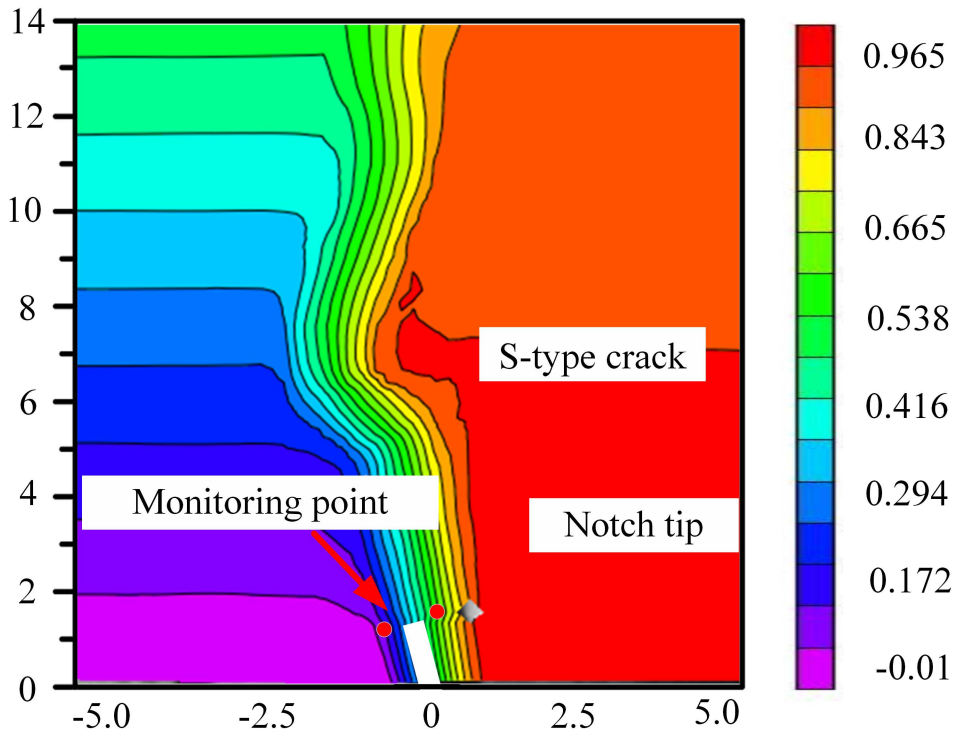

Supplement: S1 File — This is the Sl File legend. (PDF) [file pone.0333227.s001.pdf]
